# A Comprehensive Review of Polysaccharide-Based Hydrogels as Promising Biomaterials

**DOI:** 10.3390/polym15132908

**Published:** 2023-06-30

**Authors:** Achraf Berradi, Faissal Aziz, Mounir El Achaby, Naaila Ouazzani, Laila Mandi

**Affiliations:** 1 National Center for Research and Studies on Water and Energy (CNEREE), Cadi Ayyad University, P.O. Box 511, Marrakech 40000, Moroccoouazzani@uca.ac.ma (N.O.); 2 Laboratory of Water, Biodiversity and Climate Change, Faculty of Sciences Semlalia, Cadi Ayyad University, P.O. Box 2390, Marrakech 40000, Morocco; 3 Materials Science and Nano-Engineering (MSN) Department, Mohammed VI Polytechnic University (UM6P), Lot 660—Hay Moulay Rachid, Benguerir 43150, Morocco; mounir.elachaby@um6p.ma

**Keywords:** hydrogels, polysaccharides, classification, synthesis, characterization, applications

## Abstract

Polysaccharides have emerged as a promising material for hydrogel preparation due to their biocompatibility, biodegradability, and low cost. This review focuses on polysaccharide-based hydrogels’ synthesis, characterization, and applications. The various synthetic methods used to prepare polysaccharide-based hydrogels are discussed. The characterization techniques are also highlighted to evaluate the physical and chemical properties of polysaccharide-based hydrogels. Finally, the applications of SAPs in various fields are discussed, along with their potential benefits and limitations. Due to environmental concerns, this review shows a growing interest in developing bio-sourced hydrogels made from natural materials such as polysaccharides. SAPs have many beneficial properties, including good mechanical and morphological properties, thermal stability, biocompatibility, biodegradability, non-toxicity, abundance, economic viability, and good swelling ability. However, some challenges remain to be overcome, such as limiting the formulation complexity of some SAPs and establishing a general protocol for calculating their water absorption and retention capacity. Furthermore, the development of SAPs requires a multidisciplinary approach and research should focus on improving their synthesis, modification, and characterization as well as exploring their potential applications. Biocompatibility, biodegradation, and the regulatory approval pathway of SAPs should be carefully evaluated to ensure their safety and efficacy.

## 1. Introduction

Based on *Grant & Hackh’s Chemical Dictionary*, superabsorbent polymer (SAP) is defined as a solid with a cross-linked three-dimensional network that swells and retains 20–95% of water due to its hydrophilic polymeric chains [1]. More precisely, superabsorbent polymers, or hydrogels, are three-dimensional hydrophilic homopolymer or copolymer networks obtained from natural and/or synthetic monomer or polymer materials, which have the potential to absorb, thanks to their cross-linked macromolecular chains, considerable quantities of water [2] or biological fluid [3], without dissolution due to their physically and/or chemically cross-linked structures [4,5].

However, SAPs have the ability to respond to environmental stimuli such as variations in temperature, pH, solvent composition, enzymes, light, and electrical fluids. This means that SAPs can exhibit various behaviors or properties in response to these external factors. For instance, changes in temperature or pH can affect the swelling or absorption capacity of SAPs. In addition, variations in solvent composition may affect their solubility, while enzymes can catalyze specific reactions or degradation of the SAPs. Light exposure can trigger photo-degradation or changes in their physical properties, whereas electrical fluids can influence their conductivity and electrical properties. All these characteristics make SAPs versatile and suitable for various applications where environmental responsiveness is desired [6,7]. 

The first SAP was synthesized in 1938, using acrylic acid and divinylbenzene thermal polymerization in aqueous media [8]. Wichterle and Lim (1960) synthesized the first generation of SAPs based on poly (hydroxyethmethacrylate) [9], used in contact lenses due to their high hydrophilicity and biocompatibility. Otherwise, traditional SAPs are based on synthetic monomers such as methacrylic acid [10], acrylic acid [11], or acrylamide derivatives [12], and other acrylates [13,14], which lead to the synthesis of synthetic hydrogels. However, although these synthetic SAPs present good mechanical properties, good durability, and high swelling capacity, they are usually poorly degradable and non-environmentally friendly. Contrariwise, natural hydrogels, based on bio-sourced polymers, possess significant advantages such as abundance, non-toxicity, biocompatibility, and, most importantly, biodegradability, which make them environmentally friendly. Moreover, among natural polymers, polysaccharides can easily form SAPs through chemical cross-linking (covalent bonds), physical cross-linking (non-covalent bonds), or a combination of both, making polysaccharides’ gelling a polyvalent and promising approach for hydrogel synthesis. 

Since environmentally friendly processes and products are of the utmost interest, many industrial investigations have been carried out into synthesizing polysaccharide-based hydrogels, demonstrating potential application in different areas due to their excellent water absorption ability, low cost, and biodegradability [15,16]. Moreover, they can be easily adapted to have the desired mechanical and chemical properties for being applied in various domains such as horticulture and agriculture [17], personal hygiene [18], water purification [19], food industries [20], and biosensors [21], and they are of great importance in the biomedical field [22,23]. 

The objectives of this critical review paper are:

- To classify hydrogels based on various factors (source, cross-linking method, polymer composition, crystallinity, electrical charge, form, and pore size);
- To provide an overview of the basic research on natural hydrogels based on chitosan, cellulose, starch, and other polysaccharides;
- To summarize the various methods of hydrogel synthesis and provide information on hydrogel characterization;
- To give a view of the various applications of polysaccharide-based superabsorbent polymers;
- To discuss the practical applications of hydrogels based on polysaccharides in various fields, including agriculture, wastewater treatment, and biomedical engineering;
- To identify the current technology’s challenges and limitations and suggest future research and development directions.

## 2. Classification of Superabsorbent Polymers

**Figure 1:**
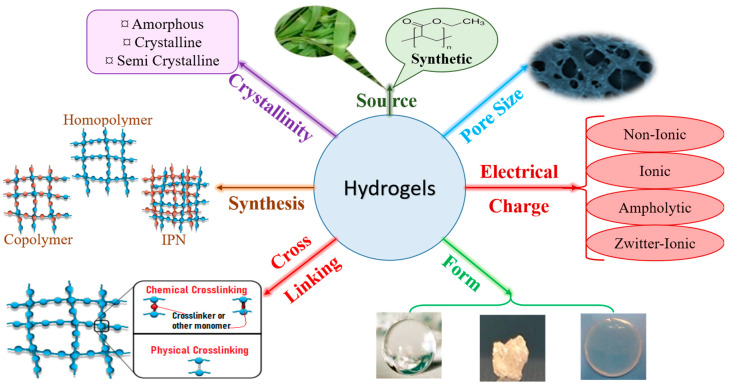
Hydrogel classification.

Hydrogels can be classified based on various properties such as their source, structure, composition, and preparation method (Figure 1).

### 2.1. Source

Hydrogels may consist of natural polymers, synthetic polymers, or a combination of both.

#### 2.1.1. Hydrogels Based on Natural Polymers

This type generally has inherent biocompatibility and biodegradability. It is formed from bio-sourced polymers that can be classed into two main groups:

- Polysaccharide-based hydrogels

Cellulose has abundant hydroxyl groups and can easily prepare hydrogels with fascinating properties and structures. Moreover, these SAPs are environmentally friendly and renewable materials with good biodegradation properties [24]. 

Chitosan, a cationic copolymer with good biocompatibility and biodegradability due to its hydrophilic character and ability to degrade, makes it a valuable source of natural SAPs. These high-potential SAPs are useful in various contexts, including medical applications and wastewater treatment [19]. 

Alginate is a widely known biomaterial and one of the most widely used biomaterials for hydrogel formation due to its biodegradation properties, biocompatibility, and non-toxic nature. It is an excellent source of hydrogels for various applications, such as wound healing [25].

- Hydrogels based on polypeptides

Collagen, the main material of the extracellular matrix of humans and animals, is an excellent source material for hydrogel synthesis, which can be used for tissue engineering and biomedical applications. For example, because of their transparency, collagen-based hydrogels may be used as a corneal alternative for corneal regeneration [26]. 

Gelatin-based SAPs (a denatured collagen product) have evolved into large polyvalent biomaterials. Gelatin is increasingly used in combination with collagen due to its high water dissolution capacity and economic productivity. Gelatin-based SAPs are also naturally biodegradable and do not present any toxicity to human cells (in medical applications) [27]. 

#### 2.1.2. Hydrogels Based on Synthetic Polymers

Synthetic hydrogels are primarily petrochemical based, generally synthesized from synthetic monomers such as methacrylic acid, polyacrylic acid, vinyl acetate, and polyethylene glycol [28]. Due to their widely variable and easily altered properties, synthetic hydrogels are widely used and studied today. In addition, it is possible to regulate the structures of those SAPs by modifying the preparation techniques or the chemical composition. These hydrogels can be applied in several areas. As suggested, they can be customized or modified to present mechanical or chemical properties adapted to a specific need while adjusting their properties, including swelling capacity, mechanical strength, stability, and porosity [29]. However, due to their synthetic nature, these polymers often cause inflammatory reactions, have low clearance rates, and do not possess good degradability and biocompatibility [29].

#### 2.1.3. Hybrid Hydrogels

Hybrid hydrogels come from a combination of natural polymers (for their biocompatibility and biodegradability) and synthetic ones (for their functionalities and mechanical properties) and are sometimes reinforced by some charges. For instance, a smart nanocomposite hydrogel can be developed from a combination of natural polymers (gelatin and chitosan) and synthetic polymer (poly N-isopropylacrylamide-co-acrylic acid) and used as an injectable drug delivery system [30]. This hydrogel was prepared through ionic interactions between gelatin and laponite particles with opposite charges, and the other polymers (chitosan and copolymer) were added to render the gel pH responsive. Composite SAPs targeted for bone tissue engineering were synthesized using poly (vinyl alcohol) and pectin as basic polymers [31]. After freezing–thawing, the mixed solution was immersed in calcium to provide physically cross-linked SAPs with high porosity, appropriate mechanical properties, and suitable cell adhesion ability. Finally, the same process was used in a (carboxymethyl cellulose/polyvinyl alcohol) eco-friendly SAP synthesis by repeating the freeze–thaw cycles at various concentrations of both polymers in each cycle [32].

### 2.2. Type of Cross-Linking

Cross-linking is the creation of a link that connects the polymeric chains, leading to a hydrogel with a stable structure. The use of cross-linking may alter the physical properties of the polymer depending on the degree of cross-linking. The SAP becomes elastic at low cross-linking rates but rigid at high cross-linking rates. Cross-linking decreases the resulting SAP’s viscosity and increases its glass transition temperature, toughness, and strength [33]. 

According to the cross-linking type, hydrogels could be physically cross-linked or chemically cross-linked [34]. Table 1 lists some characteristics of hydrogel cross-linking methods. The main methods of cross-linking types are shown in Section 3.2. 

### 2.3. Polymer Composition (or Network Nature)

The number of monomers alters the type of hydrogel and gives SAPs with different properties. According to their polymer composition, SAPs can be divided into three types:

- Homopolymeric hydrogels: Their network is made from a single species of monomer, which serves as the network’s basic component [35]. This monomer can be cross-linked according to its nature and the polymerization process;
- Copolymeric hydrogels: Its network comprises two or more distinct monomers, at least one of which is hydrophilic, arranged in a random configuration, sequenced or alternated along the polymeric network’s chain [36];
- Interpenetrated polymer network (IPN) hydrogels: They consist of two independently bonded natural and/or synthetic polymers arranged as a network, where just one is cross-linked. They are synthesized by immersing a pre-polymerized hydrogel in a monomer solution in the presence of an initiator [37]. In addition, these hydrogel systems have better fracture toughness with maximum compressive stress than traditional hydrogels, owing to the ability of one network to maintain the SAP’s elasticity. Another ability is to self-heal when the charge is removed, such as for the SAP prepared from elastic chemical cross-links and self-healing physical cross-links formed together to ensure entanglement [38].

### 2.4. Crystallinity (Network Morphology)

Superabsorbent polymers acquire several crystalline structures through manufacturing based on the employed technique. The hydrogels can be classified, according to the morphology of the network, as amorphous, crystalline, or semi-crystalline [39,40]. 

### 2.5. Electrical Charge or Ionic Particles

The polymeric chain of some SAPs contains charged particles and is conductive. SAPs may be classified into four classes according to the availability of electrical charge in their chains [41]:

- Non-ionic;
- Ionic (anionic or cationic);
- Amphoteric (ampholytic) electrolyte, containing acid and basic groups;
- Zwitterionic, containing anionic and cationic groups in each repetitive unit.

### 2.6. Form

Superabsorbent polymers can achieve many expected shapes depending on the requirements and polymerization technique applied. They can be in the form of microspheres, film, balls, matrices, etc.

### 2.7. Pore Size

Pores can form in hydrogels either by separating phases during synthesis or as smaller pores in the network. Therefore, medium size, size distribution, and pore interconnections are crucial factors in an SAP’s matrix [42]. Thus, SAPs can be classified according to pore size, and they have been separated into superporous (pore size > 100 μm), macroporous (pore size: 0.1–1 µm), microporous (pore size: 0.1–0.01 μm), and nanoporous (pore size: 0.001–0.01 μm). 

## 3. SAPs Based on Polysaccharides: Synthesis and Types

### 3.1. Polysaccharides

Generally, there are six major natural polymer macromolecules: proteins, polynucleotides, polysaccharides, polyesters, polyisoprenes, and lignin [43]. In this review, we are interested in polysaccharides, representing a broad class of carbohydrate polymers with monosaccharide units linked through glycosidic links. 

They play significant functions in biological processes [44,45]. Therefore, they are very suitable for preparing SAPs owing to their excellent properties, such as hydrophilicity, non-toxicity, renewability, high swelling ability, biodegradability, biocompatibility, and possible chemical modifications. 

All these properties and functionalities make polysaccharides “ecologically friendly” products for various applications. Furthermore, polysaccharides can be divided into two groups (Table 2): homopolysaccharides, which consist of the same monosaccharide, while heteropolysaccharides are heteroglycans with distinct monosaccharides.

There are three categories of polysaccharides depending on their source:

- Animal polysaccharides: Divided into glycosaminoglycans (such as hyaluronic acid, heparin, and keratan sulfate) and chitin/chitosan. They comprise many functional groups such as -NH_2_, -OH, -COOH, and -SO_3_H;
- Plant polysaccharides: Generated from plant cell metabolites. The most abundant are starch and cellulose;
- Microbial polysaccharides: Produced by many bacteria such as *Pseudomonas elodea* and *Sphingomonas paucimobilis*.

Table 3 lists the most well-known polysaccharides depending on their source, with their characteristics, and gives some examples of hydrogels prepared from extracted polysaccharides. 

### 3.2. Methods of Preparing Natural SAPs

**Figure 2:**
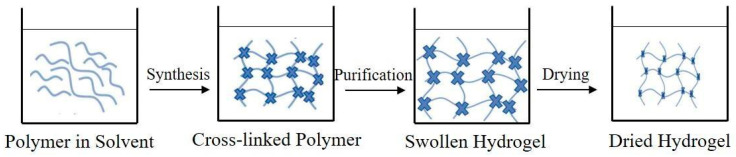
Schematic representation of general hydrogel preparation steps.

An SAP can be developed by various techniques depending on the desired properties and purpose. The general method of its synthesis is shown in Figure 2.

#### 3.2.1. Physical Cross-Linking: Reversible Hydrogels

Physically cross-linked SAPs are formed through molecular interactions and maintained by transient low-energy cross-linking nodes ranging from 1 to 40 kJ/mol [78]. However, their thermodynamic and mechanical state acts directly on the number and strength of the cross-linking nodes. The interactions are reversible (associations break and continually re-form) and may be affected by modifying environmental conditions or applying mechanical stresses. In addition, the reversible property provides the characteristic of links that can be modulated with the deterioration of environmental conditions [79]. 

For example, alginate can be cross-linked by ionic interactions using calcium ions (Ca^2+^) to form a hydrophilic three-dimensional network that gives an egg-box model structure. Each calcium cation is coupled with the carboxyl and hydroxyl groups of four G-monomers from two adjacent polymeric chains [80]. 

Table 4 below summarizes the most investigated methods of physical SAP synthesis.

#### 3.2.2. Chemical Cross-Linking: Permanent Hydrogels

**Figure 3:**
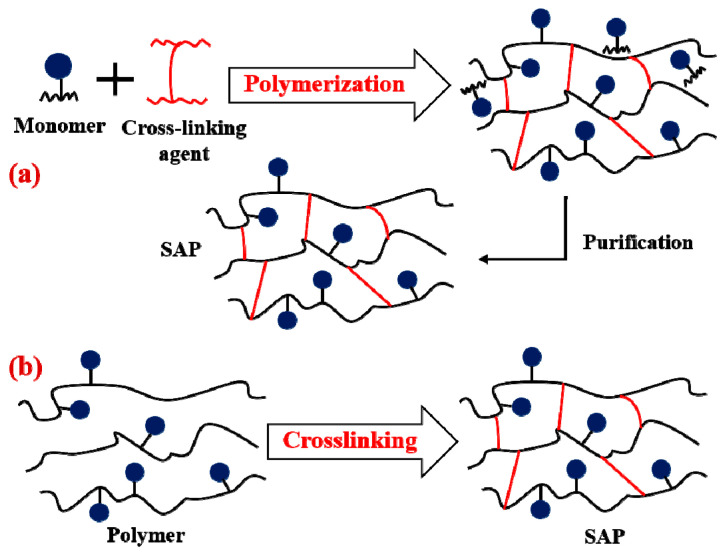
Hydrogel synthesis: (**a**) Three-dimensional polymerization; (**b**) direct cross-linking of water-soluble polymers.

Chemically cross-linked SAPs consist of polymer chains bound together by covalent bonds [95] of high energy between 150 and 900 kJ/mol [78]. The preparation of these hydrogels can generally be achieved in two ways. The first is through the polymerization of a hydrophilic monomer with a cross-linking agent (three-dimensional polymerization, Figure 3a), and the second is through direct cross-linking of water-soluble polymers (Figure 3b) [96]. 

Unlike physical SAPs, chemical ones tend to be more chemically stable with robust mechanical properties and permanent due to the strong covalent bonds between polymeric chains, making them suitable for long-term use [79]. 

Polysaccharide-based chemical SAPs can be achieved by reacting their functional groups (such as COOH, OH, and NH_2_) with a cross-linker in the presence of other reagents, ensuring that the desired functional group builds a network between the bonds to make the SAP. So, using a cross-linking agent leads to forming a 3D structure, improving mechanical properties and stability by affecting physical properties such as the polymer’s viscosity, elasticity, and insolubility [97]. The cross-linking agents most commonly used are N,N’ methylenebisacrylamide [98], epichlorohydrin [99], glutaraldehyde [100], poly(vinyl alcohol) [101], ethylene glycol dimethacrylate [102], and poly(ethylene glycol diacrylate) [103]. Table 5 groups the most common methods used to prepare permanent natural hydrogels.

### 3.3. Cellulose-Based Hydrogels

As known, cellulose is the Earth’s most prevalent polysaccharide, and it is a sustainable alternative for developing SAPs with various properties for many applications. Recently, cellulose-based hydrogels were synthesized for drug release in cancer therapy [125]. SAP films (PCNCHFs) were prepared from carboxymethyl cellulose (CMC) and polyvinylpyrrolidone (PVP) and reinforced with sepiolite nanoclay (0, 0.3, 0.5, 0.7, 0.9, and 1.5%) by thermal treatment followed by a casting process. 

The developed hydrogels were characterized to investigate their structural (via FTIR, XRD), morphological (via SEM, EDX), and thermal properties (via DSC, TGA). 

FTIR analysis confirmed the SAP’s formation. Moreover, it has been reported that the reinforcement of SAPs resulted in low crystallinity, higher tensile strength, and thermal stability because of the strong interfacial interaction between functional groups of sepiolite silanol and CMC. In addition, PCNCHFs showed higher swelling capacity with good pH sensitivity. The increase in pH led to an increase in the drug’s release percentage, which could be suitable for drug delivery [125]. 

Many SAPs made from cellulose nanofibers that act as reinforcement in the polymer matrix were synthesized by graft polymerization into some synthetic polymers, such as polyacrylamide [126]. However, a nanocomposite IPN hydrogel, based on cellulose nanofibers (CNFs) and acrylic acid (AA), was synthesized through radical polymerization using MBA as cross-linker and KPS as initiator, with different urea/AA ratios, after diluting CNFs and neutralizing AA with aqueous KOH solution in order to raise pH and accelerate polymerization [127]. The SAP’s formation was confirmed by FTIR analysis, and it was found that AA’s chemical interaction with urea and CNFs changed the amorphous structure of SAPs. In addition, the monomer content affected the hydrogel swelling properties. Incorporating urea and CNFs generates compact hydrogels with high cross-linking density, reducing absorption capacity. Still, introducing urea in the hydrogel resulted in a more extended network with higher swelling capacity, but an excessive amount (at 10/10 of the urea/AA ratio) resulted in a decrease in absorption capacity. 

Graphene oxide (GO) was utilized as an enhanced component to synthesize cellulose-based hydrogels, cross-linked by MBA [128]. They were prepared by mixing cellulose with MBA and GO (dispersed in H_2_O) in NaOH/urea aqueous solution with different ratios. The prepared cellulose/MBA/GO gel showed a high water retention ratio (3.22 − 3.16 × 10^4^%), good transparency (25% before swelling SAPs and 35–65% after swelling), improved mechanical properties (28–45 KPa and 40–59 KPa, respectively), and good texture properties (maximum of adhesiveness: 4.2941 N.mm). In addition, GO reinforced the hydrogel’s network because of the formation of hydrogen bonding between GO and cellulose. However, the prepared SAPs with good recoverability showed good adsorption rates, with maximal adsorption of methylene blue (138 mg/g). The adsorption of Cu^2+^ was improved due to the porous structures of SAPs and to some polar groups (C=O, -OH) that interact with Cu^2+^ via electrostatic interaction. 

Conversely, regarding physical hydrogels, there are many reversible hydrogels based on cellulose, such as one formed via ionic interaction where glycine (deprotected amine groups) plays a cross-linker role [129]. Cellulose and glycine are dissolved in a NaOH alkali solution and then neutralized with acetic acid. The gel’s formation was confirmed by FTIR and Raman spectroscopies. As a result, CL5Gly30 (with 30% *w/v* glycine) has the highest water absorption capacity, which can absorb water up to seven times its dry weight because of its porous 3D network structure confirmed via SEM images. Moreover, all prepared hydrogels show good mechanical and thermal properties. 

CMC is an anionic polysaccharide easily cross-linked by polycations, such as Ca^2+^, Al^3+,^ and Fe^3+^. For instance, pH-sensitive bionanocomposite hydrogel beads [130], based on CMC and Halloysite nanoclay–atenolol drug (HNT-AT), were synthesized by the same method in [131]. The CMC/HNT-AT was synthesized through a coordination bond between Fe^3+^ ions and -COOH groups on CMC, where these groups convert, in neutral and basic conditions, to carboxylate anions and electrostatically interact with Fe^3+^ cations to form gels. FTIR confirmed the formation of CMC/HNT-AT beads, and HNT nanoclay improved the beads’ morphological properties, as confirmed by SEM and XRD techniques. The prepared SAPs showed good thermal and swelling properties, and the nanoclay increased the beads’ thermal stability. AT release results in different pH and the increase in the pH leads to an increase in the drug-release rate due to the fast dissolution of atenolol molecules adsorbed onto HNT surfaces. Those beads had low water absorption capacity compared to CMC-AT ones because HNT increases the cross-linking density (HNT acts as a cross-linking agent, interacting with CMC’s functional groups), which reduces the amount of released AT drug [130]. 

### 3.4. Chitosan-Based Hydrogels

After cellulose, chitosan is the most abundant polysaccharide widely used to make chemical and physical hydrogels. 

Concerning chemically cross-linked chitosan-based hydrogels, two bio-based modifiers, nanographene oxide (nGO) and genipin (GP), were employed to improve the properties of a chitosan (CS)-based composite SAPs, utilized for medical applications [132]. GP is the cross-linking agent and nGO was derived from chitosan via microwave-assisted carbonization (via sulfuric acid) and oxidation (by nitric acid). The SAPs, with different compositions, were prepared by mixing CS, GP, and nGO solutions, which are obtained by dissolving CS in acetic acid, GP in ethanol, and nGO in deionized water. The nGO and GP amounts influenced the hydrogel properties including surface wettability, rheology, swelling rate, and diclofenac sodium (DCF) adsorption. The composite SAPs had a porous structure and rough surface, and this roughness was independent of GP content but increasing the nGO amount led to increased surface roughness. As a result, good absorption capacity results were found, which decreased where GP (cross-linker) or nGO (acting as a cross-linking catalyst) amount increased in the gel due to increased cross-linking density. In fact, the two modifiers increased the DCF’s adsorption ability due to some secondary interactions and surface wettability. To date, several chitosan/GO-based SAPs have been synthesized for adsorption applications, such as the adsorption of heavy metals (such as Cr(VI)) [133], organic compounds [134], and pharmaceutical wastes [135]. 

Besides chitosan/GO-based hydrogels, there are several chitosan-based SAPs used to prepare adsorbents owing to their high adsorption capability through active sites for metal adsorption (amino and hydroxyl groups). So, many were prepared to serve as wastewater treatment systems by adsorbing heavy metals and pollutants, such as oil [136], Cr (VI) [137], cadmium [138], Cu^2+^ and Co^2+^ [139], or dyes such as Acid Blue 9 and Allura Red [140]. 

For example, cadmium and methylene blue were highly and successfully adsorbed by a chitosan/magnetite-based hydrogel [141]. This hydrogel achieved high methylene blue and cadmium sorption capacities of 23.478 mg/g and 80.383 mg/g, respectively. FTIR and thermogravimetric analyses confirmed the interactions between the hydrogel matrix and pollutants. The prepared SAPs were synthesized by adding magnetite nanoparticles to a hydrogel previously prepared by dissolving chitosan in an acetic acid solution and cross-linked by MBA [141]. 

Another chitosan-based hydrogel was prepared for wastewater treatment, using glutaraldehyde as a cross-linker for bonding iron oxide nanoparticles (Fe_3_O_4_) with chitosan before grafting poly (acryloyloxyethyltrimethyl ammonium chloride) (PDAC) onto a chitosan backbone [142]. PDAC was introduced to offer reactive quaternary ammonium sites for the adsorption of Cr^6+^ and an organic dye. The prepared Fe_3_O_4_-CS/PDAC hydrogels exhibited good and high adsorption capacities, with a maximal adsorption capacity of 163.9 mg/g for Cr^6+^ and 762.2 mg/g for sunset yellow, in addition to good reusability, which is higher than 90% removal efficiency after five cycles of adsorption and regeneration. 

Some chitosan-based hydrogels were prepared by graft polymerization of some vinyl monomers onto chitosan, such as acrylonitrile [143], acrylamide [144], and acrylic acid [145]. For example, a chitosan–graft–acrylic acid has been synthesized by free radical polymerization and has the potential to be a water-retaining agent [146]. The chitosan was converted to amino ethyl chitosan (AMECS) by dissolving chitosan powder in 2-chloroethylamine hydrochloride solution before the graft polymerization. Confirmed by FTIR and ^13^C NMR, AA was effectively grafted onto the amino ethyl chitosan’s backbone after mixing AEMCS with AA, using ammonium persulfate (APS) as initiator and MBA as cross-linker agent. The synthesized hydrogel could be expected to have an IPN structure, considering -COOH of AA can react with -NH_2_ and -OH on the chitosan derivate. The prepared hydrogels had an increased thermal stability network and better mechanical strength and displayed high water absorption capacity and salt resistance. 

Among chitosan-based chemical hydrogels, this animal polysaccharide, with a pair of unshared electrons, can make physical SAPs by forming a hydrogen bond [147] or ionic interaction [148,149,150] with anionic compounds via electrostatic interactions. 

Chitosan-based physical hydrogels were prepared via electrostatic interaction between -NH_3_^+^ groups of CS and the phosphate groups (PO_4_^3−^) of the anionic cross-linkers [151]. As a result, the prepared SAPs had a hydrophilic surface after cross-linking by sodium hexametaphosphate (SHMP) and sodium tripolyphosphate (STPP). Moreover, the pore size was reduced when the cross-linker concentration increased, and the hydrogel structure became more compact. The hydrogels showed a pH-responsive character, where the swelling capacity decreases with increasing pH when the cross-linker content is lower than 1% *w*/*v*, because of the electrostatic interactions’ charges, and increases at 1% *w*/*v* cross-linker. In addition, the cells’ viability was affected by the cross-linker concentrations’ variation, and it decreased where the SHMP and STPP concentrations increased. The STPP-cross-linked chitosan showed higher cell viability with better swelling capacity than SHMP-cross-linked chitosan. 

CHCAUR was developed using chitosan, citric acid, and urea in a 1:2:2 weight ratio via hydrothermal synthesis [152]. The cross-linking reaction occurred due to chitosan’s protonation via the trifunctional acid, causing a physical cross-linking by ionic bonds. The increase in the viscosity of the medium and gelation occurs because of that reaction. CHCAUR was extremely porous due to gas evolution (NH_3_ and CO_2_) as urea reacted with citric acid, forming urea citrate adduct. Otherwise, the SEM images exhibited that 0.359 mm was the median pore diameter of the CHCAUR and the standard deviation was 0.134, where porosity was 27.61%. CHCAUR absorbed a maximum of about 1250 g/g of distilled water. In addition, with a nitrogen content of 11%, this SAP can be exploited as a polyvalent material in agriculture, particularly as a slow-release agent of nutrients in the soil. 

### 3.5. Starch-Based Hydrogels

Much focus has been placed recently on developing starch-based SAPs for application in various fields. 

As a coating, starch-based SAPs could be used in slow-release fertilizers. The nutrient’s release rate depended on the microstructure, the durability, and the water absorption rate of the prepared SAPs, which was influenced by the reaction’s condition or the degree of saponification [153]. For instance, starch is transformed to carboxymethyl starch (CMS) by mixing corn starch with an aqueous solution containing NaOH and monochloroacetic acid for three hours at 60 °C, to synthesize a controlled-release fertilizer network (P-CMS-g-PAM) based on phosphate-bound carboxymethyl starch–graft–polyacrylamide, to steadily deliver phosphate fertilizer to the plant [154]. Using MBA and KPS, the hydrogels were synthesized by grafting AM onto phosphorylated starch with various fertilizers. The optimal grafting of AM could be reached at 0.5 mol/L of KPS and 0.8 mol/L of the monomer concentration. However, P-CMS-g-PAM with a high phosphate to CSM ratio (1:0.66) showed the highest swelling percentage and exhibited 87% phosphorous release on the 30th day. 

Besides fertilizer coatings, many starch-based hydrogels have been used as a coating for blood-contacting devices [155] and food ingredients [156]. For instance, a starch-based nanosorbent hydrogel was synthesized for coating derived from nanoscale spherical biochar using glycerol as a cross-linker agent for Cr(VI) and naproxen drug removal [157]. The optimum percentage mass ratio between starch and nanoscale biochar for preparing SAPs was 2% (*w*/*w*). The evaluated hydrogels were characterized by high swelling capacity (500%) and exhibited great stability for ten cycles using HCl (0.1 mol/L) for regeneration. However, they were good nanosorbents for Cr(VI) (97.55%) and drug (90.07%) removal after five cycles. 

IPN hydrogels used as a soil conditioner were produced through irradiation cross-linking between starch, polyvinylpyrrolidone (PVP), and acrylamide (AM). In different ratios, the free radical polymerization was induced by gamma radiation (at 30 kGy) to prepare PVP/PAAM, PAAM/ST, and PVP/St IPN hydrogels [158]. Increasing starch content in the hydrogels decreases their swelling due to increased inter- and intra-molecular hydrogen bonds in the hydrogel network. Therefore, after application trials, the influence of hydrogels was very important in the growth of plants in this order: PVP/PAAM > control > PVP/St > PAAM/ST. 

Other than acrylamide, many hydrogels were prepared and showed good properties via grafting starch onto some synthetic polymers, such as polyvinyl alcohol [159,160], polyacrylic acid [161], and furamic acid [162]. In addition, grafting acrylic acid to starch is among the common techniques used to improve the water absorption capacity of those SAPs. For example, a novel modified water reservoir hydrogel has been synthesized via solution polymerization of sulfamic acid-modified starch and acrylic acid, cross-linked with MBA [163]. As a result, the swelling ratios of the prepared SAP were 1026 g/g in deionized water and 145 g/g in 0.9 wt% NaCl solution. In addition, 2-acrylamido-2-methyl-1-propanesulfonic acid (AMPS) was added to a cassava starch–acrylic acid mixture to enhance the cross-linking density and the storage modulus of prepared St-g-AA-AMPS hydrogels [164]. Increasing the AMPS amount in the gels increases the swelling rate until reaching a maximum of 1200 g/g and 90 g/g in distilled water and 0.9 wt% NaCl solution, respectively, at an AA/AMPS molar ratio of 8.5/1.5, before decreasing. This is due to the high hydrophilicity of AMPS, which improves the swelling capacity and salt tolerance of SAPs. 

### 3.6. Composite Hydrogels

Table 6 shows some polysaccharide-based composite hydrogels, i.e., composed of more than one polysaccharide:

### 3.7. Hydrogels Based on Other Polysaccharides

In addition to the polysaccharides discussed above, there are many other polysaccharides used to synthesize superabsorbent hydrogels, such as alginate [175,176], pectin [177], salecan [178,179], konjac glucomannan [180], etc. 

For instance, sodium alginate was oxidized by periodate to make alginate dialdehyde (ADA). ADA was complexed with borax, followed by a self-cross-linking reaction with gelatin via a Schiff’s base reaction, for developing a chemical hydrogel for medical purposes [181]. The prepared hydrogels (15ADA15G, 15ADA20G, and 20ADA15G) were developed with different compositions of borax (0.025, 0.05, and 0.075, 0.1 M) and gelatin (15 and 20% p/v) and gelling time decreased with increasing borax concentration. The injectable 15ADA20G hydrogel showed better mechanical properties (295 KPa in compressive strength), with 423 ± 20%, whereas 15ADA15G had the maximum water uptake (514%). All prepared SAPs showed good cytocompatibility and good integration. 

Guar gum-based SAPs used for the agricultural purposes were synthesized by grafting guar gum (GG) with acrylic acid, then cross-linking with ethylene glycol di methacrylic acid (EGDMA) using benzoyl peroxide as an initiator [182]. The hydrogels showed improved thermic stability and a spongy surface with many interspace voids. Furthermore, they were found to have a 77-day half-life and were proven biodegradable through soil burial biodegradation studies. In addition, the swelling test was performed at different pH to investigate the impact of pH and the concentration of different components on the swelling capacity. As a result, the hydrogel prepared with EGDMA (0.5 mM/g of GG), BPO (0.25 mM/g of GG), and AA (150 mM/g of GG) was the best, and the maximum water-absorption capacity was 806 g/g. 

Additionally, without grafting on acrylate, guar gum was cross-linked with borax, with different compositions, for developing a sorbent hydrogel used for water purification [183]. As a result, the SAP prepared with 20% (*w*/*w*) borax reached the maximum water absorbency. The water purification was confirmed by a dye removal test of aniline blue, with a maximum decolorization of 94.30% within 50–60 min. 

Alginate is the most used polysaccharide to prepare physical hydrogels [184,185]. For instance, calcium ion was used as a cross-linker to develop sericin–sodium alginate-based hydrogels for wound dressing [186]. This semi-IPN hydrogel exhibited effective antibacterial properties, moisture retention properties, and excellent cytocompatibility, where the animal experiment showed a maximum of 99% wound contraction ratio after 12 days, showing that the prepared SAP can effectively promote wound healing. 

Concerning pectin, a PC-g-(SA-co-NIPA))-IPN hydrogel was synthesized by solution polymerization of sodium acrylate (SA), N-isopropylacrylamide, and pectin, using MBA as a cross-linking agent and sodium bisulfite/potassium persulfate as a redox pair of initiators [187]. First, SA was cross-linked via a free radical polymerization reaction; then, the initiators were added to perform hydrogel synthesis under a nitrogen atmosphere. The prepared hydrogels were applied as adsorbents of dyes (MV and MB) and metal ions (Cu^2+^, Pb^2+^, Zn^2+^, and Co^2+^) from aqueous solution, where the pollutant’s adsorption capacity increased following the order: Zn (II) < Co (II) < Cu (II) < Pb (II) < MB < MV, with a maximum of 50.01, 51.72, 54.86, 137.43, and 265.49 mg/g, respectively. 

## 4. Bio-Based SAP Characterization

### 4.1. Gel Fraction Study 

Several methods have been proposed to determine a hydrogel’s gel content. 

The first one estimates the gel fraction by measuring the insoluble part in a dried specimen after being immersed in distilled water for 16 h [188] or 48 h [189]. The gel fraction percent is determined as follows:

(1) $$\%\;Gel\;content=\frac{W_{1}}{W_{0}}\times 100$$

The initial weight of the dried SAPs before immersing in water is *W*_0_ and *W*_1_ is the weight of dried samples after removing the soluble part. 

In another method, to determine the gel fraction in methyl hydroxyethyl cellulose-based SAPs [190], the dried SAPs were put into a 4 × 4 cm sieve before immersing in hot distilled water (80 °C) for 1 h, to eliminate soluble impurities. 

Afterward, those SAPs were immersed for 15 min in ethanol to eliminate organic impurities. Finally, they were oven-dried for two days at 60 °C, and the gel content was determined using the following formula:

(2) $$\%\;Gel\;content=\frac{W_{d}-W_{s}}{W_{i}-W_{s}}\times 100$$

where *W_d_* corresponds to the dried SAP’s weight after extraction, *W_s_* represents the sieve’s weight, and *W_i_* is the dried SAP’s initial weight. 

The degree of gelation varies according to several parameters, such as the amount of polymers or dose irradiation in the case of SAP synthesis by irradiation. Some examples of gel fraction studies are summarized in Table 7. 

### 4.2. Structural Analysis

#### 4.2.1. FTIR

As we know, Fourier transform infrared spectroscopy (FTIR) is widely used to identify polymers’ chemical structure, measure vibrational energy transitions, and give details on several atoms and chemical bonds involved [193]. So, the use of FTIR for hydrogel’s characterization involves studying the molecular structure of SAP via absorption spectra [194]. 

The NaCMC hydrogel synthesis was confirmed via FTIR analysis, based on the presence of the same peaks that appeared on the spectrum of the starting material, in addition to an extra peak at 1751 cm^−1^ due to the electrostatic interaction between -COOH groups of CMC and Fe^3+^ [192]. 

#### 4.2.2. NMR

Several nuclear magnetic resonance (NMR) modes (NMR-H, NMR-C, and pulsed field gradient NMR) were used in hydrogel characterization. The change in the spectral lines of the polysaccharide’s structure after preparing the SAP to confirm the cross-linking was successful and that the hydrogel was prepared [195]. 

For instance, NMR-H analysis was used to confirm the formation of a chitosan-based SAP named CHCAUR based on the spectral lines of CHCA and CHUR [152]. Fang et al. used NMR-C analysis to confirm the formation of a chitosan derivative (AEMCS) and prepared chitosan-based SAPs (CS-SAP and AEMCS-SAP) [146]. However, pulsed gradient NMR spectroscopy is useful for characterizing SAPs used as drug delivery systems. 

#### 4.2.3. XRD

X-ray diffraction (XRD) is widely used to identify the crystalline and amorphous phases in hydrogels while providing the percentage of crystallinity in the hydrogel matrix. Furthermore, the creation of a cross-linked network could also be verified by XRD [196]. 

XRD analysis was used by Nie et al. to follow the structure occurring within the cellulose-based hydrogel beads after adding calcium ions with different concentrations, proved by an increase in the intensity of the peaks at around 22° when the concentration of the calcium solution increased [197]. In addition, 70% calcium solution was found to be the optimum concentration for preparing beads with improved crystallinity. 

#### 4.2.4. UV–Vis

Ultraviolet and visible absorption spectrometry (UV–Vis) is a quantitative analytical method based on the absorption of radiation by molecules in a clear solution, giving information about the substance’s concentration in the solution. 

Using this technique is an attractive option, especially for evaluating drug-release or nutrient-release mechanisms when using hydrogels for drug administration (such as chloramphenicol [198]) or slow-release fertilizers [199] and also calculating the absorption capacity of pollutants for wastewater treatment, such as in the removal of diclofenac sodium from pharmaceutical wastewater [132]. 

#### 4.2.5. Raman

Raman is a non-destructive molecular spectroscopy technique [200], where the hydrogel’s molecules are irradiated by radiation. Once they are excited, they return to their original state by emitting a photon of different energy which is recovered by the detector and compared with the original signal. For instance, this was used to see calcium ions’ effect on the cellulose structure in SAP beads, and it was proved by the presence of new peaks at 554, 527, and 481 cm^−1^ [197]. 

### 4.3. Morphological Analyses

#### 4.3.1. SEM

Scanning electron microscopy is a morphological analysis that can achieve spatial resolutions to image the characteristic “network” structure in hydrogels, as it can provide information on surface topography, morphology, and porosity. For instance, SEM images revealed that cellulose-based semi-IPN SAP had a porous surface and large surface area [201]. In addition, cellulose beads physically cross-linked with calcium ions showed various morphological properties that depend on the amount of CaCl_2_, where all prepared SAPs showed a nanoporous structure, and it was found that 70% CaCl_2_ was the optimum concentration for good morphological properties [197]. 

Starch-based hydrogel has a porous structure, which is improved by the entanglement of PVP and PAAm matrices [158]. 

However, when the network structure is intimately linked to a large amount of water, it is difficult to avoid the artifacts caused by dehydration. Environmental SEM (ESEM) has a clear advantage in allowing the study of hydrogels in the presence of water [202]. 

#### 4.3.2. AFM

Atomic force microscopy (AFM) is used to obtain a 3D topographical image with a high resolution of hydrogels. It can image both dry and wet SAPs. The AFM images of a cellulose-based physical SAP (NaCMC) revealed that when the percentage of cellulose and the ionic cross-linker increases, the cross-linking density increases, which leads to a smoother surface of SAP and a decrease in the surface roughness parameters [192]. 

### 4.4. Mechanical and Thermal Analyses

#### 4.4.1. Thermal Analysis

The thermal properties of hydrogels can be characterized by thermogravimetric analysis (TGA) and differential calorimetric analysis (DSC) during their decomposition. They can also help to examine their exothermic (heat release) or endothermic nature (heat absorption) and give information about SAPs’ thermal stability. 

TGA measures the variation in the weight or mass of an SAP subjected to a high temperature. This could result in a mass loss (vapor emission, decomposition, loss of volatile matter) or a mass gain (gas fixation, oxidation, etc.). 

Based on TGA curves of some cellulose-based SAPs [54], the residual lignin fragments attached to the cellulose increase the thermal stability of prepared SAPs because lignin is more resistant to thermal degradation than cellulose. In addition, DSC curves revealed that the glass transition temperature of those SAPs increases with increasing citric acid. In addition, TGA and DSC could also determine free and bound water present in SAPs, such as for chitosan SAP [203]. 

#### 4.4.2. Mechanical Analysis

Natural SAPs have lower mechanical resistance than synthetic ones. However, the mechanical properties are a crucial point in the study of hydrogels and make it possible to relate these systems’ great structural relations/properties. 

For instance, the cross-linker agent’s amount could impact the SAP’s mechanical properties, such as for a CMC-based SAP which shows an increase in its network rigidity when the concentration of the citric acid increases [204]. The same behavior was observed in the composite SAP based on alginate and chitosan and reinforced by zinc oxide nanoparticles as they improved the mechanical properties of the prepared SAPs, thanks to their interactions with polysaccharide chains via H-bonding and electrostatic interactions [166]. 

Dynamic mechanical analysis (DMA) and rheology are the two commonly used techniques to characterize the mechanical properties of SAPs.

##### Dynamic Mechanical Analysis

DMA is used to study the viscoelastic or thermo-rheological properties of hydrogels [205], depending on the buffering or oscillatory frequency, determining the modulus of elasticity and damping values after application of an oscillating force (deformation or stress) on the sample [206]. 

For instance, DMA was used to investigate the mechanical properties of bacterial cellulose-based hydrogel composites synthesized via gamma-ray irradiation [207]. The SAP’s tensile strength increases when the irradiation dose increases until 40 kGy and decreases at irradiation of 50 kGy. Therefore, the irradiation dose of 40 kGy was chosen as the optimum dose for synthesis, with a high value of tensile strength (46.3 MPa) and a strain value of 36.6%.

##### Rheology

Rheology studies the flow and deformation of a material under the effect of applied stress through a rheometer. Hydrogels can show viscoelastic behavior and their macroscopic mechanical properties can be determined according to their structure. 

In the case of a hydrogel solution studied at low frequencies, the solution has a viscous behavior (*G″* > *G′*), whereas an elastic behavior (*G″* < *G′*) dominates in the case of high frequencies, where G’ is the conservation module and G’ is the loss module. In addition, it is possible to distinguish chemical hydrogels from physical hydrogels through mechanical spectra [208,209]. In particular, in the case of chemical SAPs, the spectrum consists of almost horizontal curves, and the *G*′ and *G″* curves are almost independent of and parallel to the frequencies, with a ratio (*G*″/*G*′) of less than 0.1 [210]. On the other hand, physical SAPs have a slightly frequency-dependent profile of modules *G*′ and *G″* with a ratio (*G*″/*G*′) greater than 0.1 [211]. 

For instance, the addition of chitosan in a cellulose/chitosan-based SAP improved its mechanical strength (critical stress and toughness), attributed to the formation of stronger H-bonds between -OH groups (of cellulose) and -NH_2_ groups (of chitosan) [168]. However, adding too much chitosan significantly decreased the hydrogel’s strength because more NH_2_-NH_2_ H-bonds were formed than OH-NH_2_ H-bonds, noting that NH_2_-NH_2_ has a lower binding force than OH-NH_2_. This technique was used to define the gelation time of an oxidized alginate-based SAP, which decreases when the amount of borax (solvent) increases, as aldehyde groups are made accessible for reaction with gelatin [181]. The optimal gelation time was 4 min at 37 °C when using 0.05 M of borax. 

### 4.5. Biodegradability

Environmental issues require using polysaccharide-based hydrogels, especially for agricultural and biomedical uses, because when released into the environment, they are degraded by microorganisms and do not impact the environment and human health. However, the SAP chemical structure, responsible for several parameters (reactivity, hydrophobicity, swelling behavior, stability) influences the biodegradation rate. As a result, natural SAPs undergo significant changes in their properties and structures after biodegradation [212]. 

The three most often used degradation tests are:

- Soil burial: An established and standardized technique where the tested SAP is buried in soil, then washed and weighed after a defined time, and the result is expressed as a weight loss percentage for a predetermined time [213].
- Enzymatic degradation: The glycosidic bonds in the polysaccharide chains are generally degradable by enzymes. These enzymes are *α-amylase*, which hydrolyzes the α-D-glucose residues in the starch chain [214], and *lysozyme*, used for chitosan-based SAP [215].

Additionally, *cellulase* was used for enzymatic biodegradation of a cellulose-based SAP [216]. Approximately 10 mg of the tested hydrogel was incubated in the enzyme solution for different durations. After washing and drying the gel under a vacuum at 35 °C, the result was expressed as a weight loss percentage through Equation (3):

(3) $$Weight\;loss\;\%=\frac{W_{0}-W_{i}}{W_{0}}\times 100$$

*W_0_* and *W_i_* are the weights of the hydrogel before and after enzyme treatment, respectively.

- Microbial degradation: Using a microbial oxidative degradation analyzer, the hydrogel is mixed with sea sand and compost, calculating the quantity of dissipated CO_2_ and producing H_2_O during degradation [217].

### 4.6. Swelling Mechanism of Hydrogels in Water and Various Parameters Affecting It

**Figure 4:**
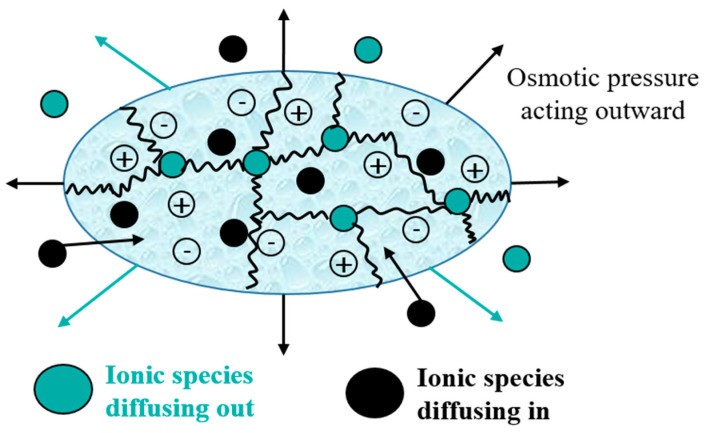
Swelling of a hydrogel.

The swelling process (Figure 4) is due to an osmotic pressure gradient. When the water diffuses towards the network, the polymer chains expand, generating an entropic elastic shrinking force due to the cross-linking nodes that increase the pressure inside the network, and when this pressure becomes sufficient to compensate for osmotic pressure, the network becomes in equilibrium with the surrounding environment, and the SAP ceases to swell [218]. 

In more detail, three crucial processes make up the lengthy process of the swelling mechanism. Firstly, the polar hydrophilic groups of SAP are hydrated by water with the formation of H-bonds, which appear as primary bound water. Next, the water interacts with the exposed hydrophobic groups, then appears as secondary bound water. Thus, the primary and secondary bound water combine to generate the total bound water. 

Finally, the cross-links prevent the network’s osmotic driving force from achieving infinite dilution, causing more water to be absorbed. The absorbed water in the equilibrium swelling is termed free water (the bulk water). 

Absorption capacity is critical for evaluating SAP properties. The pre-weighing dry samples are submerged in distilled water. Then, they are measured at different time intervals after removing excess surface water. Afterward, with these values, the swelling percentage may be obtained. 

The various methods most used for measuring the swelling capacity of SAPs are: 

Method X: Based on the Japanese Industrial Standard *K8150* method. It consists of immersing the dried SAP in distilled water at room temperature for 48 h with magnetic stirring. Once the hydrogel reaches the swollen state, it is filtered through a 30-mesh stainless steel net (681 μm). Then, the swelling rate is determined by Equation (4):

(4) $$Swelling=\frac{W_{s}-W_{d}}{W_{d}}\times 100$$

where *W_d_* and *W_s_* are the weight of dried and swollen gels, respectively [219]. 

Method Y: Based on the dispersion, using a volumetric vial of the dry SAP (0.05–0.1 g) in 20–30 mL of water at room temperature for 48 h. Then, the mixture is centrifuged to acquire the hydrated SAP layers. Afterward, the unabsorbed water is eliminated, and the swelling rate can be measured according to method X above with the same Equation (4). 

Method Z: Based on the Japanese Industrial Standard *K7223* method. The dried SAP is immersed in distilled water at room temperature for 16 h. Then, it is filtered through a stainless-steel net of 100 meshes (149 μm). The swelling rate is measured using Equation (5):

(5) $$Swelling=\frac{Z}{Y}\times 100$$

where *Z* represents the weight of the dried SAP and *Y* corresponds to the weight of the insoluble water-extractable fraction [188]. 

Tea bag: It is the most practical, quick, and appropriate technique for limited amounts of samples (0.1–0.3 g). The dry hydrogel (weighed *M*_0_) is stored in a tea bag (acrylic/polyester gauze with fine mesh), then the bag is plunged into an excessive quantity of water for 60 min to attain the equilibrium swelling [199]. The tea bag is weighed (*M_1_*) after removing the excess solution by hanging the bag until no liquid drops off. The swelling capacity is calculated by Equation (6):

(6) $$Swelling=\frac{M_{1}-M_{0}}{M_{0}}$$

After determining the swelling rate, it is better to draw the curve of the swelling-time profile, which is a graphical illustration of the swelling rate over time and is acquired by measuring the sample swelling at consecutive time intervals. 

For instance, the swelling-time profile of a cellulose/carboxymethylated chitosan-based SAP revealed that the prepared SAP using 25% carboxymethylated chitosan had a higher absorbance of distilled water (610 g/g) [220]. 

**Figure 5:**
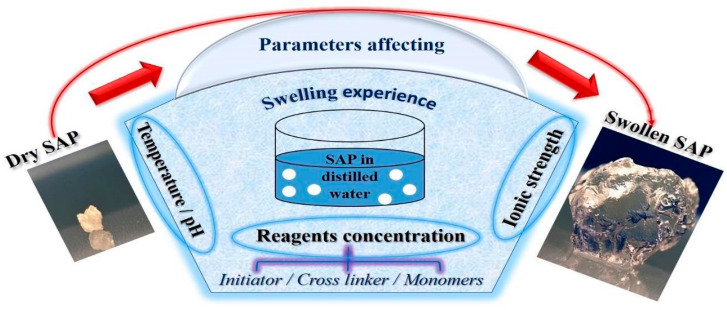
Factors affecting absorption capacity.

The absorption capacity of bio-based hydrogels depends on many factors that should be considered during swelling studies (Figure 5). 

#### 4.6.1. Reagents’ Concentration Effect

##### Effect of Initiator Concentration

The initiator plays a meaningful role in the hydrogel synthesis by generating numerous active sites on the monomer. So, an increase in initiator concentration results in a large number of free radicals, which leads to more cross-linking density in the network, affecting the swelling ratio. 

Generally, most hydrogels undergo a variation in their swelling capacity as a function of the initiator concentration, in the form of an increase with initiator concentration followed by a decrease after an initiator quantity, defined as an inflection point in the curve. 

For instance, the effect of initiator concentration on the swelling capacity of a hydrogel, based on guar gum (GG) grafted with acrylic acid (AA) and cross-linked with ethylene glycol di methacrylic acid (*EGDMA*), has been studied with the initiator being benzoyl peroxide (BPO) [182]. 

The quantities of BPO used were 0.1, 0.125, 0.25, 0.5, and 1 mM/g of GG. Keeping EGDMA concentration fixed, the concentration of BPO was varied to study its effect on water absorption capacity. However, the swelling capacity increased to a maximum of 625 mL/g at a BPO concentration of 0.25 Mm (inflection concentration). This is due to the slow breakdown of BPO at lower concentrations, which stops the formation of the hydrogel network because of the slow polymerization reaction with lower initiator quantities, giving less swelling at a lower concentration. In addition, at a BPO concentration beyond 0.25 mM, the swelling capacity decreased when increasing its concentration. This is due to the high cross-linking density in the SAP network, which reduced the SAP’s available free volume.

##### Effect of Cross-Linker Concentration

Superabsorbent hydrogels may have a swelling rate of around 100–1000 g/g, which is inverse to the cross-linking density [221]. So, a higher cross-linking rate leads to greater rigidity of the three-dimensional structure. The mobility of polymeric chains is reduced, resulting in decreased swelling capacity. The same behavior was noticed in some polysaccharide-based hydrogels, which were chemically cross-linked with citric acid [165], self-cross-linked [167,181], or cross-linked via ionic interaction with FeCl_3_ [192]. 

The cross-linking density of GG-AA-EGDMA [182] was found to be a crucial element that regulates the swelling of SAPs. The swelling capacity decreased with increasing cross-linking density after recording a maximum of 0.5 mM of EGDMA. That decrease could be due to either insufficient cross-linking, which leads to partial dissolution of SAP in solution, or a decrease in the SAP’s strength because of a weak cross-linking. Additionally, 0.5 mM of EGDMA was considered an optimum level for the SAP synthesis because it has maximum swelling.

##### Effect of Monomer Ratio

The swelling capacity depends on monomer concentrations and their hydrophilic character. For instance, carboxymethyl cellulose (CMC) cross-linked with epichlorohydrin maintains the swelling capacity and is affected by the mass ratio of CMC [222]. The swelling ratio increases in a composition range of CMC of 2–3% (wt), while it decreases beyond 3%. That could be explained by increasing the hydrophilic polymer (CMC) content in the hydrogel, increasing the affinity for water, resulting in a higher swelling capacity at 3% CMC content. In addition, at more than 3% CMC, it falls because of the increase in the network chain density, which decreases the diffusion of water molecules and reduces the macromolecular relaxations. Similar results have been obtained on CMC-based hydrogel [192] and other polysaccharide-based SAPs [191]. 

Furthermore, the impact of changing the acrylic acid ratio in GG-AA-EGDMA hydrogels on absorption capacity was studied [182]. At less than 150 mM of acrylic acid (AA), with increased content, polar -COO^−^ groups undergo better intra- and inter-molecular cross-linking in addition to electrostatic repulsion, which leads to an increase in swelling capacity. However, a further increase in the AA amount beyond 150 mM/g showed a significant decrease in the swelling capacity due to increased homopolymer content. Additionally, some hydrogels show simple behaviors when increasing the monomer content, which causes a reduction in swelling capacity due to the increase in cross-linking and rigidity of their networks, such as of cellulose [167]. 

#### 4.6.2. Temperature Effect

Temperature is a critical parameter that can positively or negatively affect the swelling ratio. It can impact the water uptake capacity of bio-based SAPs, which display sensitivity of their swelling to temperature change in the inflating medium. 

For instance, nanoporous sodium carboxymethyl cellulose (NaCMC) beads were sensitive to pH and temperature variations [192]. 

The swelling percent of NaCMC hydrogels increased slightly when the temperature increased from 10 °C to 60 °C. This behavior can be explained by improving polymer chains’ thermal mobility. In fact, when the temperature increases, the thermal mobility of polymer chains and the relaxation of their structure increase, and as a result, the swelling increases. However, some hydrogels could lose more than 50% of their capability to absorb water when heated at different temperatures, such as the semi-IPN SAP based on poly ([N-tert-butyl acrylamide]-co-acrylamide) and hydroxypropyl cellulose [223], which exhibits, contrary to the previous NaCMC hydrogels, a negative temperature-sensitive property, that is, swelling at a lower temperature and shrinking at a higher temperature. This is because the volume phase transition temperature of that SAP is also regarded as the temperature at which the phase separation ratio is the highest or the temperature at which the swelling ratio of the SAP decreases most dramatically. 

#### 4.6.3. pH Effect

pH-responsive polysaccharide-based hydrogels are an issue of interest in recent research [182,183]. Ionizable polymers having a *pKa* value between 3 and 10 are commonly considered suitable candidates for pH-responsive bio-SAPs [224]. For instance, this type of SAP’s major in vivo use controls the release of medicines into specific organs where the environment changes because of pathological circumstances or intra-cellular compartments [225]. The swelling capacity of these bio-sourced SAPs varies with pH due to the formation of ions at specific pH values, at which their functional groups can repel or attract each other. 

Generally, when the pH of the outer solution exceeds the *pKa* value of the acid groups, the SAP becomes polyanionic. In contrast, the SAP becomes polycationic when the pH is under the pKb value of the basic groups. 

On the one hand, for polysaccharide-based hydrogels that contain anionic groups (such as -COOH or -SO_3_H) [182,191], the swelling capacity increases with increasing pH. 

**Figure 6:**
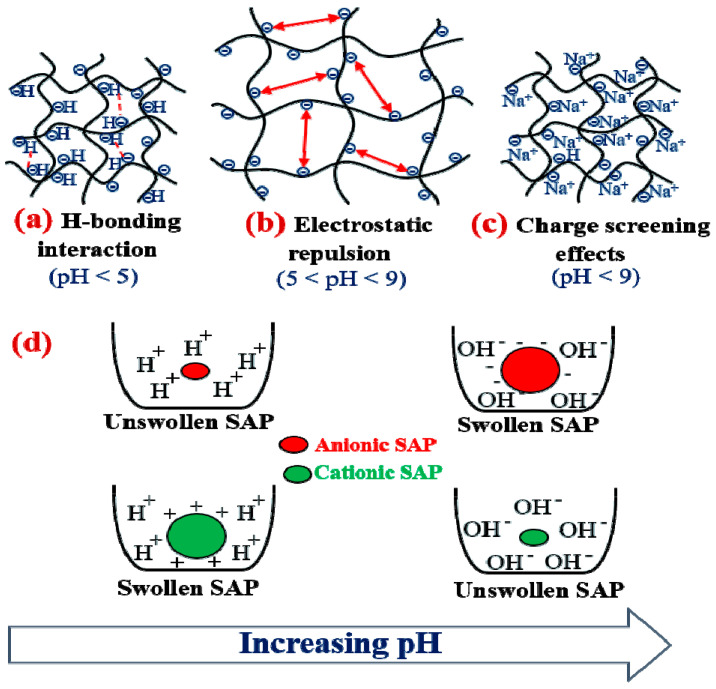
Structure models of anionic hydrogels at various pH (**a**–**c**); swelling behavior of anionic and cationic SAP at various pH (**d**).

Under acidic conditions, at pH < *pK*a, the -SO_3_H or -COOH groups stay protonated, forming H-linking interactions between these groups and generating physical cross-linking between the hydrogel macromolecular chains. As a result, the expansion of the SAP network is restrained, causing a reduction in swelling capacity (Figure 6a). 

However, increasing pH (pH > *pK*a), H-bond disruption occurs in basic media. As a result, the acidic groups dissociate, increasing the concentration of anionic groups (–COO^−^ or –SO^−3^), reinforcing anion–anion repulsions causing chain expansion, and increasing the swelling capacity (Figure 6b). 

However, in very basic conditions (pH > 8–12), some researchers [226] have described a decrease in swelling rate related to the “*charge screening effects*” of excess counter ions (such as Na^+^) in the swelling media (Figure 6c). 

On the other hand, the hydrogels with cationic groups have a reverse response to pH variations (Figure 6d). They swell at low pH and shrink at higher pH. For example, the swelling capacity of a chitosan-based hydrogel remained stable within the pH range of 7 to 11, with no significant differences. However, when the pH increased from 11 to 12.5, the swelling capacity decreased from 38.5 ± 0.5 g_water_/g_hydrogel_ (at pH 11) to 12.4 ± 0.3 g_water_/g_hydrogel_ (at pH 12.5) [227]. 

Additionally, in the case of hydrogel that possesses anionic and cationic groups from the basic polymers, such as a magnetic and pH-sensitive hydrogel derived from *κ*-carrageenan and carboxymethyl chitosan [228], under acidic conditions, the anionic carboxylate (-COO^−^) and neutral amine groups (-NH_2_) in the carboxymethyl chitosan are converted into a neutral carboxylic acid (-COOH) and cationic ammonium (-NH^3+^) groups, respectively. Furthermore, the sulfate group (−OSO_3_^−^) on *κ*-carrageenan causes ionization at low pH (due to *pK*a < 2). However, an ionic complex between -NH^3+^ and −OSO_3_^−^ is immediately formed under acidic conditions due to the remaining sulfate groups. 

#### 4.6.4. Ionic Strength Effect

Ionic strength is a complex aspect that can positively or negatively influence the absorption ability of bio-SAPs. 

Studying the ionic strength effect on the swelling rate of SAPs is generally performed using various salts (ionic compounds), namely KCl, NaCl, LiCl, MgCl_2_, CaCl_2_, and AlCl_3_. The measurements can be compared by using a dimensionless salt sensitivity factor given by Equation (7):

(7) $$f=1-\frac{S_{saline}}{S_{water}}$$

*S*_saline_ and *S*_water_ are the swelling capacity in saline solution and water, respectively. ***f*** indicates the saline effects, i.e., when it is near 0, the ionic effect is low; when it is near 1, the salinity effect is strong [229]. 

The swelling rate of bio-based SAPs depends on saline solution concentration, the charge of ions, and ion sizes, which are three sensitive factors that can impact an SAP’s swelling rate (Table 8). Before dissecting these three factors, it should be noted that the swelling degree is managed by the balance between the osmotic pressure (which results from the mobile ion concentrations between the interior of the hydrogel network and the external immersion solution) and the elastic response of the hydrogel. If the osmotic pressure decreases, the hydrogel’s volume decreases, leading to the gel’s shrinkage and causing a decrease in the swelling rate.

### 4.7. Loading and Release of Nutrients

A few uses of these eco-friendly hydrogels in agriculture, such as for the slow release of nutrients, are based on the SAP’s ability to load and release such substances to the plants as required. 

The hydrogel’s capacity to load a solute (such as urea or potassium nitrate) as a function of time can be measured by Equation (8):

(8) $$Loading\;\left(\%\right)=\frac{S_{0}-S_{t}}{S_{t}}\times 100$$

where *S*_0_ corresponds to the initial concentration of nutrients before contact with the SAP and *S_t_* represents the residual concentration of nutrients after a time *t* of hydrogel immersion in the solution. 

Moreover, loading (%) could be calculated by Equation (9), based on the weights, after immersing dry hydrogel into an aqueous solution of nutrients (e.g., urea) for 12 h or 24 h and drying the swollen hydrogels at room temperature for 3 days:

(9) $$Loading\;\left(\%\right)=\frac{W_{1}-W_{0}}{W_{1}}\times 100$$

where *W*_0_ and *W*_1_ are the weights of unloaded and loaded dry SAPs, respectively [232]. 

In turn, the percent solute release is calculated by Equation (10):

(10) $$Releasse\;\left(\%\right)=\frac{R_{t}}{L}\times 100$$

where *R_t_* is the total amount of solute released at time *t* and *L* is the initial solute concentration loaded in the SAP. 

Numerous polysaccharide-based hydrogels with good loading/releasing capability have already been developed, such as a cellulose-chitosan hybrid hydrogel which was capable of releasing nitrogen, phosphorus, and potassium slowly in soil, with a full release of around 90% after 60 days, in comparison with free fertilizers without hydrogel that achieves a full release after 5 days [233].

## 5. Applications of Polysaccharide-Based Superabsorbent Polymers

**Figure 7:**
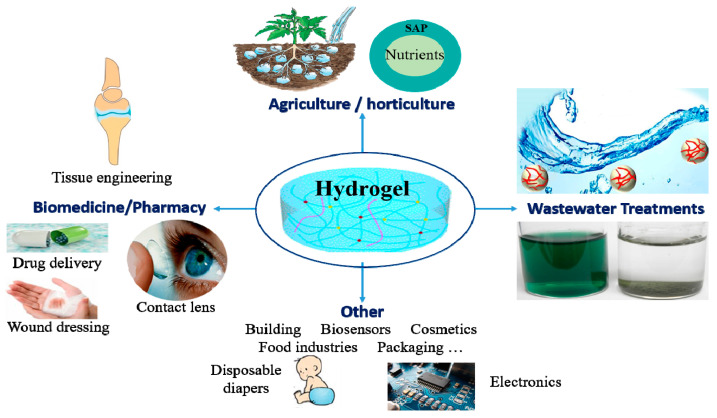
An overview of the application of polysaccharide-based hydrogels.

Figure 7 highlights the potential uses for polysaccharide-based hydrogels in diverse fields, including agriculture and horticulture, wastewater treatment, biomedicine, pharmacy, food industries, hygiene products, cosmetics, and construction biosensors. 

### 5.1. Agriculture

Water plays an essential role in agricultural production. However, water shortage leads to droughts, which cause desertification and salinization of soils. Therefore, it impacts the sustainable development of agriculture and food security, mostly in arid and desert regions, where scarcity of water resources is a serious issue. 

For that reason, enhancing water-use efficiency is of great importance to the agricultural and horticultural sectors via searching for a good solution to reduce water usage. 

In this regard, there is an increased value in using superabsorbent polymers as water-retaining agents in order to reduce water consumption, which leads to maintaining soil moisture and reducing irrigation frequency, thanks to their considerable capacity for water absorption and retention [234]. Furthermore, the swollen SAP can slowly release the uptaken water (or nutrients), which leads to maintaining soil humidity for a long time. 

**Figure 8:**
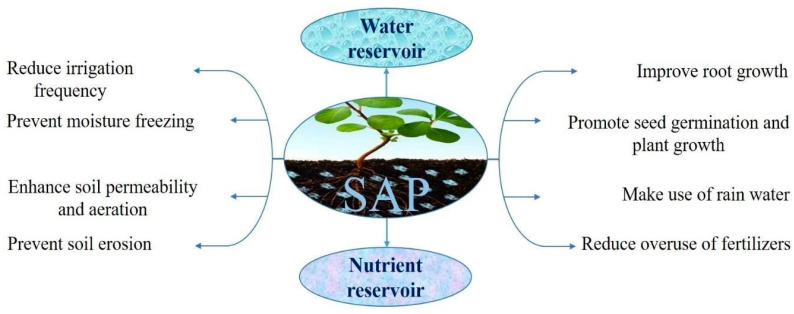
Different applications of hydrogels in agriculture.

In the agricultural sector, hydrogels are polyvalent (Figure 8).

The simplest way to use SAPs in farming is their spreading, in the dry state (powder, granules, or beads), into the soil around the plant’s roots. The efficiency of that process depends upon the properties of the soil, including aeration, temperature, water uptake capacity, and nutrient transportation. However, SAPs can also be loaded with nutrients (such as urea) and/or plant pharmaceuticals before application on the soil. After the SAP absorbs water, it releases nutrients to the plants as needed, maintaining soil moisture over extended periods. Moreover, the large-granule SAPs perform better than fine granules through the enhanced aeration of the soil. However, it is important to know that overdosing on such materials may be potentially dangerous and should be prevented. It is thus critical to determine the optimal SAP amount before applying it to the soil. 

Due to their significant swelling ability compared to polysaccharide-based hydrogel, various synthetic polymer-based SAPs, such as acrylic acid and polyacrylamide, have been extensively applied for agricultural and horticultural purposes as water reservoir agents or as a coating for slow-release fertilizers. 

Despite their high absorption capacity, these synthetic SAPs are poorly degradable, toxic, and non-environmentally friendly. By contrast, polysaccharide-based SAPs have major benefits such as non-toxicity, biodegradability, biocompatibility, availability, and low-cost production, which make them an attractive, durable alternative to synthetic ones, and applicable in agriculture and horticulture. Several polysaccharide-based hydrogels have been employed to manufacture SAPs applicable in agriculture, such as cellulose [235], starch [236], or guar gum [237], among others. 

#### 5.1.1. Water Reservoir

**Figure 9:**
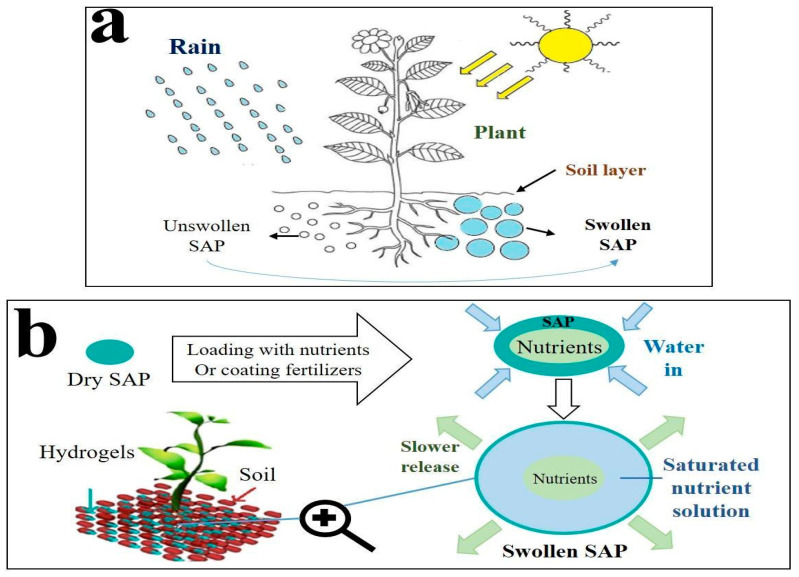
(**a**) Hydrogels as water reservoir system and (**b**) slow-release fertilizers.

Polysaccharide-based SAPs can store water in their cross-linked network, then release it slowly into the soil (Figure 9a). 

For example, chitosan-based SAPs acted as water-retaining agents in cultivation, were pH sensitive, and could retain 71% of water after one day, with a maximum water absorbency (550 g/g) in basic conditions (pH 8) [146]. 

Chitosan was copolymerized with sodium alginate and polyacrylamide through gamma rays to prepare new superabsorbent polymers applicable in maize cultivation as water reservoir agents and soil conditioners [191]. The grain yield was increased by 50% after treating maize plants with those SAPs due to the presence of two polysaccharides (chitosan and alginate), which led to acceleration and activation of some biological activities of plants, including metabolic, enzymatic, and photosynthetic capability, in addition to their hydrophilic structures that help them to absorb huge quantities of water. 

Moreover, gamma radiation was used for cross-linking starch with other polymers to be applied as a water reservoir system [158]. As a result, the sunflower growth rate was significantly influenced based on the capability to store water and release it slowly into the soil in the long term. 

Song et al. applied an alginate-based hydrogel to save and release water and essential nutrients in tobacco cultivation [238]. Their experiment succeeded because of the high water absorption and retention capacities of SAPs, and the plants grew even after stopping irrigation. Sodium alginate was the basic natural material for preparing a water-saving nanocomposite SAP through free radical graft polymerization of alginate, acrylic acid, acrylamide, and rice husk ash, which increased water absorption capacity from 830 g/g for SAP without rice husk to 1070 g/g after adding it [226]. The prepared SAP could be an efficient water management system for agriculture. 

Besides the polysaccharides mentioned above and based on cellulose derivatives, carboxymethyl cellulose is widely used for making hydrogels for agricultural applications. For instance, a chemically cross-linked carboxymethyl cellulose-based hydrogel was prepared to be applied in tomato cultivation to improve its growth rate by economizing water use [230]. The dried SAP powder was applied in red soil near the tomato plant roots with various amounts of hydrogel to study the impact of SAP/soil ratio on the water absorption capacity. The prepared hydrogel absorbs more water when its concentration increases in the soil, and then this water reservoir material releases water slowly to the plants as needed. 

In contrast, for the combination of two hydrophilic polymers, carboxymethyl cellulose was copolymerized with starch to make hydrogel with high water retention capacity [239]. The resulting hydrogel released water for the plants for a long time. The results revealed a 50–70% increase in soil water retention capacity after applying the SAP with different concentrations. 

Salmawi et al. reinforced a carboxymethyl cellulose-based hydrogel with clay montmorillonite to improve its water absorption capacity, making it a successful water-managing material, especially in dry areas [240]. 

Song et al. grafted hydroxyethyl cellulose on the copolymer of acrylate and acrylamide to have a cellulose-based hydrogel with a maximum water absorption capacity of 240 g/g [241]. The prepared SAP was mixed with soil to improve its water-holding ratio, which increased from 29.68% for the untreated soil to 55.69% for the soil containing SAP. 

Moreover, after 14 days of testing, the untreated soil lost all of its absorbed water, while the soil treated by SAP retained 14.83%. 

Finally, potassium sulfate (K_2_SO_4_) was blended in hydrogels based on methylcellulose and hydroxypropyl cellulose to induce the hydrophobic interaction between those two cellulose derivatives [242]. The physically prepared SAPs were temperature responsive and could serve as water storage for cultivation thanks to their high capacity to absorb water. 

The addition of K_2_SO_4_ improved the swelling rate and increased mechanical properties of hydrogels, and the treatment with 0.5% SAP significantly boosted the water absorption and water retention capacities of sandy soil by a maximum of 71.2%, which helped to improve plant growth by reducing the drainage overflow water below the root zone wherever potassium fertilizers were loaded into the hydrogels, meaning they are able to act as controlled-release devices in agriculture. 

#### 5.1.2. Slow/Controlled-Release Fertilizers

Global demand for commercial fertilizers is gradually increasing due to their important purpose in improving crop quality, maintaining soil fertility, and increasing yield because of their ability to release nutrients (such as nitrogen (N), phosphorus (P), and potassium (K)) to the plant. Unfortunately, their intensive uses have been associated with different types of pollution (such as N_2_O emission) caused by nutrient losses (leaching and volatilization) because of their low efficiency of nutrient uptake by plants in the environment. To overcome all these problems, many polymers have been used in agriculture as coating resin of fertilizers to achieve slow release of nutrients. In this case, the polymer acts as a physical barrier to slow down the release rate of nutrients and reduce their losses in the environment. 

However, in order to improve yields without compromising the environment, polysaccharide-based hydrogels can be a good alternative in this field. So, polysaccharide-based SAP can be impregnated or loaded (in situ loading (while processing) or post-loaded (after SAP processing)) with fertilizer compounds (urea, potassium ions, a soluble phosphate, etc.) [243], or they are used as coatings for fertilizers to develop slow-release or controlled-release fertilizer systems [244]. These environmentally friendly fertilizers reduce environmental pollution caused by nutrient losses and increase fertilizer-use efficiency by releasing nutrients into the root zone as required. 

After fertilizer coating or SAP loading, the nutrient-release stage occurs through the swelling mechanism of hydrogels while irrigating. Water penetrates the hydrogel and dissolves the nutrients inside the SAP, increasing the osmotic pressure, which leads to a slow release of nutrients through the hydrogel pores into the soil. Figure 9b illustrates the slow-release fertilizer steps. 

Starting with the most abundant animal polysaccharide, chitosan-based superabsorbent hydrogel was used as a sustainable solution for slowly releasing urea after loading [152]. The results showed that with 11% urea content in the gel, this material could be applied in agriculture as a controlled release system of micro- and macronutrients in the soil in order to improve plant growth and decrease water evaporation rate significantly due to its higher water absorption capacity of 1250 g/g. 

For plant polysaccharides, a controlled-release phosphate system was synthesized by graft polymerization between carboxymethyl starch and polyacrylamide before loading with two phosphate fertilizers [154]. The resulting SAP delivered the phosphate nutrients to the plants and achieved 87% cumulative nutrient discharge on the 30th day.

Based on the most abundant polysaccharide on Earth, Qiao et al. developed a double-coated slow-release urea fertilizer via encapsulating nitrogen granules with ethyl cellulose as the inner layer, reinforced by a second layer based on starch–polyacrylamide. The result showed a typical release behavior of nutrients after 96 h [245]. 

Graft polymerization was carried out to prepare another cellulose hydrogel as a coating for slow-release NPK fertilizers [246]. The hydrogel was prepared via graft polymerization of sulfonated carboxymethyl cellulose with acrylic acid in the presence of nutrient compounds and showed a slow-release behavior. 

Another polymerization method for making SAPs is irradiation, and Raafat et al. prepared one for agricultural uses through irradiation cross-linking between carboxymethyl cellulose and polyvinylpyrrolidone [232]. The slow-release fertilizer system was obtained by loading urea into the obtained SAP, which has a significant swelling rate of 144 g/g in addition to a good water-holding capacity based on swelling experiments (preserves 50 wt% of water after 24 h at 25 °C), to provide nitrogen nutrients for plants. In deionized water, discharged urea increased as the SAP load percentage increased and reached a maximum of 4 g/L after 5 h for 269% urea loading. 

Cassava starch and acrylonitrile copolymerization have been used to prepare a grafted starch-based SAP as the film for coating urea [247]. After application on soil, the result shows that the nitrogen was slowly and fully liberated for a long time (108 days), compared to the uncoated urea, which was completely released after 28 days. 

In addition, starch was also grafted with acrylamide and cross-linked with MBA to prepare a starch-based SAP with a high maximum water absorption capacity of 253 g/g [248]. The prepared hydrogel was used as a coating for urea fertilizers, and it was discovered that less than 15% of nitrogen was delivered after 24 h. In contrast, the release percentage was more than 80% after 40 days, showing nutrients’ slow-release behavior. 

In summary, the main advantages of applying polysaccharide-based SAP in agriculture are acting as a water or/and nutrient reservoir for the slow release of water or/and nutrients to the plant for a long time, maintaining soil humidity, and increasing soil porosity to provide better oxygenation for plant roots, which are beneficial for making use of rainwater, reducing irrigation water demand, and of course, increasing agricultural yields, as well as promoting better microbial actions in soil because of their biodegradability. 

### 5.2. Wastewater Treatment

Among various methods for wastewater treatment, such as electrochemical processes, coagulation–flocculation, ion exchange, reverse osmosis, membrane filtration, treatment with ozone, activated sludge, and biological treatment, adsorption treatment is more popular because of its convenience, facile recovery, and ease of application [249]. 

Therefore, polysaccharide-based hydrogels have been abundantly used for wastewater treatment due to their physicochemical properties, chemical stability, high adsorption ability, high reactivity, and selectivity toward pollutants. 

Table 9 lists examples of polysaccharides used to make adsorbent hydrogels for wastewater treatment. 

### 5.3. Biomedicine

Polysaccharide-based hydrogels have recently been used as an alternative to synthetic ones in biomedical and pharmaceutical applications (Table 10) because of their biodegradability, non-toxicity, biocompatibility, good water retention ability, and temperature/pH response properties. They are commonly used for drug delivery, wound dressing, and tissue engineering, as mentioned below [279,280,281]:

Drug delivery: Polysaccharide-based superabsorbent polymers offer significant advantages as carriers for active ingredients in tablet or capsule formulations, enabling sustained drug release. The process begins by loading the SAP-based hydrogel with drugs, including pesticides, nucleotides, and proteins. When the hydrogel comes into contact with body fluids, it undergoes swelling due to its superabsorbent properties. As the hydrogel swells, the incorporated drugs dissolve within the gel matrix. The hydrogel’s swelling facilitates the drug’s dissolution, which is followed by a controlled and long-term release of the drug molecules. This sustained drug release mechanism ensures a gradual and prolonged delivery of the active ingredients over an extended period of time. By utilizing polysaccharide-based SAPs for drug delivery, several benefits are achieved. First, the sustained release of drugs allows for improved therapeutic outcomes as it maintains a constant drug concentration within the body. This can be particularly advantageous for medications requiring long-term treatment or a narrow therapeutic window. Second, using SAPs as carriers enables the protection and stabilization of the drugs, preventing their degradation or inactivation before reaching the target site. This enhances the bioavailability and effectiveness of the drugs. Lastly, the biodegradable nature of polysaccharide-based SAPs ensures that they are broken down and eliminated from the body over time, minimizing any potential long-term accumulation or adverse effects. Overall, polysaccharide-based SAPs provide a promising platform for drug delivery, offering sustained release, enhanced drug stability, and biodegradability. These properties make them valuable tools for improving pharmaceutical treatments’ efficacy, safety, and patient compliance.

Wound dressing: SAPs may be specifically designed to promote wound healing and protect against infections. Natural hydrogels, including those based on polysaccharides, have been extensively researched for their application in wound healing due to their exceptional properties. These hydrogels have a high water retention ability, ensuring the wound remains adequately hydrated. They are also known for their purity and biocompatibility, minimizing the risk of adverse reactions. One key advantage of using natural hydrogels for wound dressings is their non-adherent nature. These materials do not stick to the wound bed, allowing for painless removal without causing any damage or disruption to the healing process. Additionally, they help maintain the optimal moisture balance in the wound, preventing excessive dryness or excessive moisture that could impede the healing process. Furthermore, natural hydrogels used in wound dressings have the ability to absorb excess toxins, such as bacteria and debris, from the wound site, promoting a clean and sterile environment for healing. They also exhibit good permeability to gases, allowing for proper oxygenation and gas exchange at the wound site, which is crucial for the healing process. Overall, using natural hydrogels, including polysaccharide-based ones, in wound dressings offers several advantages, including non-adherence, moisture regulation, toxin absorption, and gas permeability. These properties contribute to effectively managing and promoting wound healing while minimizing discomfort and complications.

Tissue engineering: This field aims to enhance or replace specific organs and tissues using engineered materials. Polysaccharide-based hydrogels are widely utilized in this domain of biomedical applications due to their remarkable mechanical properties, high biocompatibility, and versatile functionality. Polysaccharide-based SAPs are essential in tissue engineering solutions by acting as scaffolds or frameworks that mimic the extracellular matrix (ECM). The ECM is the natural support structure surrounding cells in tissues. By emulating the ECM, SAPs provide a conducive environment for the growth and organization of cells, facilitating the engineering of new tissues within the body. The mechanical properties of polysaccharide-based SAPs, such as their strength, flexibility, and elasticity, contribute to the structural integrity of the engineered tissues. These SAPs can be tailored to match the specific mechanical requirements of different tissues, such as bone, cartilage, skin, and muscle. Moreover, the biocompatibility of polysaccharide-based SAPs ensures that the body tolerates them well and does not trigger adverse immune responses. This is crucial for successful tissue integration and long-term functionality. In tissue engineering, polysaccharide-based SAPs offer multifunctionality, allowing for the incorporation of bioactive molecules, growth factors, or drugs to enhance tissue regeneration. They can also provide controlled release of these bioactive agents, promoting cell proliferation, differentiation, and tissue remodeling. Overall, the exceptional mechanical properties, biocompatibility, and multifunctionality of polysaccharide-based SAPs make them valuable components in tissue engineering applications. They play a pivotal role in fabricating engineered tissues, enabling the development of advanced solutions for organ and tissue regeneration in areas such as bone, cartilage, skin, muscle, and beyond. 

### 5.4. Other Applications

Finally, apart from the three previous fields, polysaccharide-based superabsorbent polymers are extensively utilized in many other applications, including: 

The food industry, where these materials can decrease fat in processed food. They are also important in the production of conventional foodstuffs as well as the development of new foods due to their good physicochemical properties. Moreover, they can act as gelling agents in the food industry [290]. Some SAPs are used to evaluate bacterial trapping and toxicity in food systems [291] and toxins and pesticides, where a cellulose acetate-based hydrogel is used to detect cholesterol based on electrocatalytic responsiveness [292].

Packaging, where these materials are used as films for packaging, such as a nitrocellulose/guar gum-based hydrogel [293], among others [294], and as paper films for food packaging to reduce the use of synthetic plastics, which affects the environment.

Due to their high liquid absorption and retention abilities, SAPs are used in disposable hygienic products, such as feminine hygiene pads, baby diapers, and adult incontinence products, to replace traditional absorbing materials, including cotton, paper, etc. [295].

Cosmetics, where these materials are used for skin care with their excellent moisturizing and antimicrobial properties, owing to their high water retention and hydrophilic properties. They also improve skin health and induce soft skin, erase fine wrinkles, increase elasticity, and strengthen skin [296].

## 6. Conclusions

Superabsorbent polymers are usually made from petrochemical starting monomers, such as acrylic ones, to make synthetic hydrogels. However, natural hydrogels, prepared from biomass such as polysaccharides, could theoretically provide environmentally friendly alternatives to synthetic SAPs. We aim to become progressively greener by replacing synthetics with bio-sourced hydrogels.

This review article discusses recent developments and advancements in polysaccharide-based hydrogel synthesis, including chemical cross-linking, physical cross-linking, and polymerization techniques, after giving a global view of the general classification of hydrogels by listing all polysaccharide properties used in SAP preparations. According to the literature review, these bio-based SAPs are known for their good mechanical and morphological properties, thermal stability, biocompatibility, biodegradability, non-toxicity, abundance, economics, and good swelling ability. The impacts of several factors, including temperature, pH, ionic forces, and reagent concentrations, on water absorption were discussed.

Easy and economically friendly polysaccharides have become the most exciting materials to convert from wealth to health. First, however, they must be ready to prepare efficient and smart hydrogels applicable in agriculture, wastewater treatment, medicine, and personal care.

## 7. Prospects

- The hydrogel field has received great attention from researchers to improve their environmental responses to promote their application in various fields. Indeed, the focus should be on natural hydrogels, biohydrogels, which are biodegradable, non-toxic, economical, and more sustainable, especially in medical fields, agriculture, food industries, and water purification systems, so as not to affect the environment and human health, while avoiding an increase in the current plastic soup caused by hydrogels based on petrochemical polymers, having a huge environmental impact.
- This type of superabsorbent polymer has many more beneficial properties than synthetic SAPs, given the economic and environmental side. However, there are still some challenges to overcome, such as limiting the formulation complexity of some SAPs, such as chitosan-based hydrogels, as it is known that chitosan is difficult to dissolve without using acids for a long time.
- The importance of extracting polysaccharides from some wastes to make hydrogels instead of commercialized ones should be recognized to reduce the product’s cost and valorize industrial wastes. In addition, incorporating waste materials into hydrogels as reinforcements can be a solution to valorize some waste materials and also improve the mechanical and adsorption/absorption properties.
- It is necessary to agree on a general protocol to be followed or to set a uniform standard for the calculation of the absorption and water retention capacity of hydrogels while defining standard conditions to be applied, such as the duration of the test, temperature, and humidity, to compare the results of one hydrogel with others.
- In the agricultural field, it is necessary to try to include swelling tests in the soil because the SAP’s ability for absorption in the soil is not as good as in laboratory-scale absorption experiments since some conditions are not controllable, such as temperature, humidity, and pH of the irrigation water.
- As synthetic hydrogels are still applied in several sectors, semi-synthetic hydrogels, known as intelligent SAPs, will require a lot of research efforts in the future, as this combination of natural and synthetic polymers will improve the durability of these synthetic hydrogels.

In conclusion, developing research on polysaccharide-based hydrogels requires a multidisciplinary approach involving chemistry, biology, materials science, and engineering. Research should focus on improving these materials’ synthesis, modification, and characterization and explore their potential applications in different fields. Additionally, hydrogels’ biocompatibility, biodegradation, and regulatory approval pathway should be carefully evaluated to ensure their safety and efficacy in different uses. Furthermore, research should explore the scalability of SAP production and investigate its regulatory approval pathway. Developing cost-effective and scalable production methods is crucial for commercialization and widespread use.

## Figures and Tables

**Table 1:** Characteristics of cross-linking methods.

	Physical Cross-Linking	Chemical Cross-Linking
Advantages	- Reversible - No need for a cross-linking agent - No need to remove the solvent’s residual amount - Excellent shear recovery (self-healing hydrogels) - Simple preparation process	- Permanent - Provides high mechanical strength - Easily approachable - Highly efficient and more controllable - Provides high molecular weight
Disadvantages	Poor mechanical strength	Need for a purification step
Techniques		
Characteristics	- Formation of non-covalent electrostatic interactions - Possibility of preparation without chemical modification of the polymers	- Formation of covalent bonds - Use of cross-linking agent - Presence of some chemical reactions

**Table 2:** Structural classification of polysaccharides.

	Unbranched	Branched	Reference
Homopolysaccharides	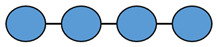	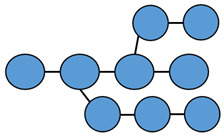	[43]
Heteropolysaccharides	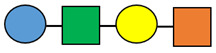	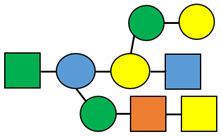	
Each of these forms below represents a different monosaccharide. 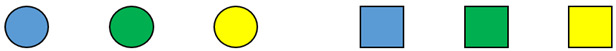 Hexose: Glc Man Gal HexNac: GlcNAc ManNAc GalNAc			

**Table 3:** Various polysaccharides most commonly used in the preparation of hydrogels, based on their source.

Polysaccharide (Subunit, Bonds)	Structure	Source	Characteristics (In Addition to Low Cost, Biodegradability, Eco-Friendliness, High Biocompatibility, Multifunctionality)	Ref.
Animal Polysaccharides				
Chitin (N-acetyl glucosamine, β1–4)	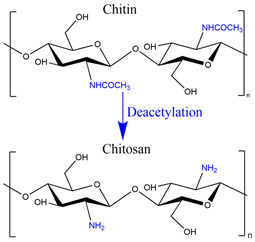	Exoskeletons of fungus, mollusks, insects, and crustaceans	- Unbranched homopolysaccharide. - The most abundant animal polysaccharide on Earth. - Present in three crystalline structures: alpha, beta, and gamma. - Renewable, with high hydroxyl, amino, and acetyl group content. - Poor solubility in solvents.	[46,47]
Chitosan (glucosamine and N-acetyl glucosamine, β1–4)		Chitin (via deacetylation)	- Unbranched homopolysaccharide. - Crystalline, cationic, and hydrophilic. - Possesses amino and hydroxyl groups. - Low solubility in many solvents, soluble in dilute acidic solutions. - Sophisticated extraction processes. - (-NH_2_) groups facilitate chemical cross-linking to make SAPs. - Its derivatives are procured via graft copolymerization, thiolation, and carboxymethylation, among other modifications. - Excellent adsorption capability. - Very viscous polymer solution.	[48,49,50]
Hyaluronic acid (D-glucuronic acid, N-acetyl glucosamine, β1–4 and β1–3)	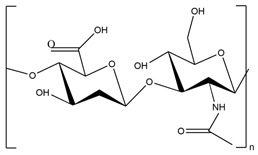	Extracellular matrix of soft connective tissues and skin	- Unbranched heteropolysaccharide. - Its solution is viscoelastic at higher concentrations. - Need for chemical modification or covalent cross-linking. - Makes chemical hydrogels. - Excellent water-holding capacity and viscoelastic properties.	[51]
Plant Polysaccharides				
Cellulose (D-glucopyranose, β-1–4)	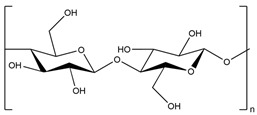	Green plants (like bamboo and trees), natural fibers, bacteria	- Unbranched homopolysaccharide. - Earth’s most abundant organic substance. - Semi-crystalline, with a high density of (-OH) groups. - -OH in positions C_2_, C_3,_ and C_6_ can serve as reactive groups for modifications, such as esterification or etherification of -OH, to produce some derivates (such as hydroxyethyl cellulose, hydroxypropyl cellulose, and carboxymethyl cellulose) for making various types of SAPs. - Difficult dissolution in water because of its crystalline regions linked by intra- and inter-molecular H-bonds. - Dissolves in organic solvents, alkali/urea aqueous medium, and ionic liquids.	[52,53,54,55,56,57]
Starch (amylose (α-1,4-linked D-glucose) and amylopectin (α-1,4- and α-1,6-linked D-glucose))	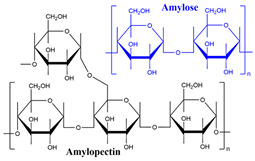	Crop seeds, potato, corn, roots, and stalks	- Branched heteropolysaccharide. - Insoluble in alcohol, cold water, or other solvents. - Composed of linear amylose (20–30%, semi-crystalline, soluble in hot water) and branched amylopectin (70–80%, highly crystalline, insoluble in hot water), with numerous hydroxyl groups. - Has a source-dependent structure. - Swells in water at ambient temperature. - Inexpensive and easy to modify with other polymers.	[58,59]
Pectin (D-galacturonic acid connected by 1→4 glycosidic bonds)	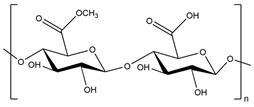	Cell walls of higher plants (e.g., black currants and apples) (Extraction with water)	- Unbranched heteropolysaccharide. - Anionic polysaccharide with hydroxyl, ester, and carboxyl groups. - Soluble in water. - Categorized according to the methoxy content: high-methoxy pectins (>50% esterified), which form gels at low pH, and low-methoxy pectins (<50% esterified), which form partially sheared gels.	[60]
Alginate (guluronic acid and mannuronic acid, β-1→4 glycosidic bonds)	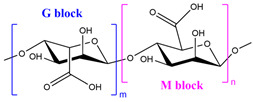	Brown seaweeds (Via treatment with aqueous alkali solutions, generally NaOH)	- Unbranched heteropolysaccharide. - Anionic polysaccharide, flexible, strong, and water soluble. - Possibility of adjusting its properties by changing the guluronic acid/and mannuronic acid ratio. - Commercially available as sodium alginate. - Makes generally physical SAPs by the addition of divalent cations.	[61,62,63,64]
Agarose (3,6-anhydro-α-L-galactopyranosyl and β-D-galactopyranosyl)	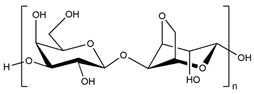	Red algae of seaweeds, e.g., *Gelidium* and *Gracilaria*	- Unbranched heteropolysaccharide. - Insoluble in cold water but soluble in hot water, forming a gel after cooling down. - Neutral and thermo-responsive polysaccharide. - Excellent water retention capability.	[65]
Carrageenan (β-(1→4)-3,6-anhydro-D-galactose and α-(1→3)-D-galactose)	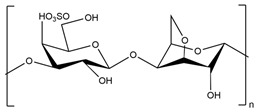 κ-carrageenan	Rhodophyceae red seaweeds	- Unbranched heteropolysaccharide. - Possesses many carboxyl and hydroxyl groups, with one sulfate group for *kappa* (κ), two sulfate groups for *iota* (ι), and three sulfate groups for *lambda* (λ) per unit. - *κ-carrageenan* and *ι-carrageenan* form stable physical hydrogels.	[66,67]
Guar gum (1,4-linked β-D-mannopyranose and 1,6-linked α-D-galactopyranose)	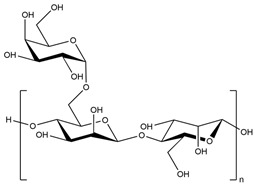	Seeds of Cyamopsis tetragonolobus	- Branched heteropolysaccharide. - Non-ionic polysaccharide. - Rapidly swells and produces viscous solution even in cold water. - Contains hydroxyl groups, which can be reactive for chemical modifications, such as introducing -COOH, -NH_2_, and -SO_3_H groups.	[68,69]
Cyclodextrin (D-glucose, α1–4-glycosidic bonds)	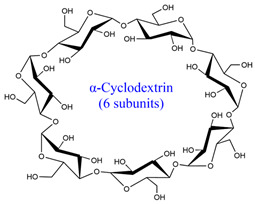	Enzymatic conversion of starch	- Unbranched heteropolysaccharide. - Cyclic structure of 6, 7, or 8 units: α-cyclodextrin (6 subunits), β-cyclodextrin (7 subunits), and γ-cyclodextrin (8 subunits). - High stability against *amylase*. - Cyclic structure with an interior hydrophobic cavity and a hydrophilic external surface.	[70]
Microbial Polysaccharides				
Pullulan (maltotriose, α-(1–6) and α-(1–4) glycosidic bonds)	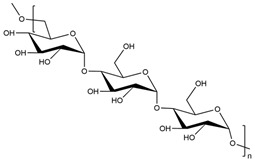	The fungus *Aureobasidium pullulans*	- Unbranched heteropolysaccharide. - Has nine -OH groups per unit, with great mechanical properties. - High chemical reactivity and water soluble. - Possibility of chemical modification (etherification, esterification, sulfonation, or oxidation) for making various hydrogels.	[71,72]
Dextran (D-glucose, α-(1–6) with branches of α-(1–3))	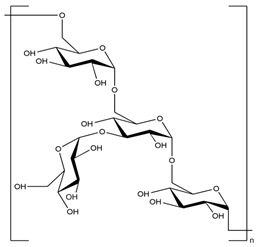	Lactic acid bacteria, e.g., *Streptococcus, Leuconostoc, Weisella, and Lactobacillus*	- Branched homopolysaccharide. - Non-ionic flexible structure due to free rotation of glycosidic bonds. - Water insoluble (with the existence of >43% of α-(1–3) linking branches), and water soluble (with 95% linear linkage). - Capable of being modified to form dextran sulfate and cationic dextran, for making diverse SAPs.	[73]
Salecan (β-1,3-glucose)	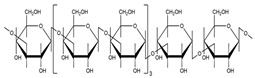	Agrobacterium sp. ZX09	- Unbranched homopolysaccharide. - Contains hydroxyl groups, soluble in water. - Has good rheological properties and forms high-viscosity solutions at low doses and shear stresses.	[74,75]
Gellan gum (D-glucose, D-glucuronic acid, and L-rhamnose)	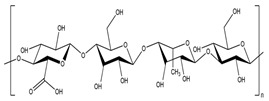	Bacteria, like *Sphingomonas paucimobilis and Pseudomonas elodea*	- Unbranched heteropolysaccharide. - Anionic and possesses many active groups: -OH and -COOH, with the possibility to obtain deacylated gellan gum by modification. - Forms physical SAPs while cationic ions such as Na^+^ and Ca^2+^ are present at low temperatures.	[76]
Xanthan gum (D-glycopyranose linked with a side chain via α-1,3 linkage)	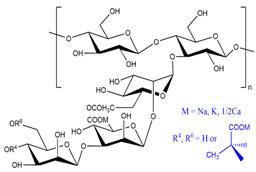	Bacteria *Xanthomonas campestris*	- Branched homopolysaccharide. - Helical structure, non-allergenic, with slow dissolution rate. - Thermo-induced behavior of its sol–gel phase transition. - Good stability at high temperatures and pH due to a dimeric or double-stranded structure. - Pseudo-plastic and non-Newtonian fluid properties.	[77]

**Table 4:** Methods of preparation of physical hydrogels.

Methods	Explanation	Ref.
Ionic interactions 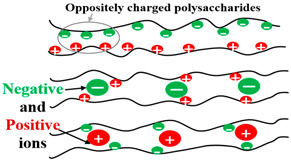	By interaction mechanism between the polymer with ionic groups and some multivalent ions (di- or trivalent) of opposite charge (counter-ions).	[81,82,83]
Hydrophobic interactions 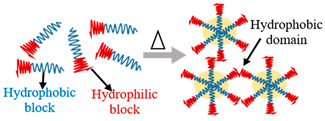	Via a free radical mechanism, a hydrophilic monomer copolymerized with a hydrophobic comonomer. Hydrophobic interactions seem strong compared to other physical interactions, such as van der Waals bonds or hydrogen bonds).	[84]
Crystallization (Freeze–Thawing) 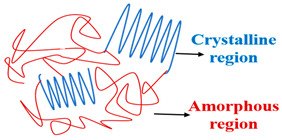	After repeated freeze–thawing cycles, the polymer acquires a phase separation, which leads to microcrystal formation in its structure, creating hydrogel. Moreover, the hydrogel’s mechanical properties may be controlled by varying cycle number, time, or temperature.	[85]
Hydrogen bonding 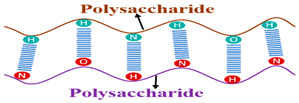	H-bonding occurs between functional groups of polysaccharides such as -NH_2_, -COOH, -SO_3_H, and -OH. The resulting SAPs are affected by several factors, such as polymer concentration, molar proportion, solution temperature, solvent type, etc.	[86,87,88,89]
Complex conservation 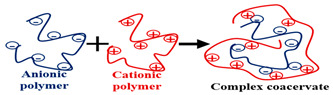	It is an association between oppositely charged polymers (polyanionic and polycationic). Opposite charge polymers attract each other, forming insoluble and soluble complexes under diverse concentrations and pH of the polymeric solutions.	[90]
Protein interaction 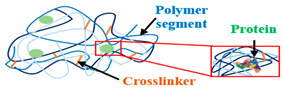	Hydrogels form by electrostatic interactions between the polysaccharide and the protein when they carry opposite electric charges.	[91]
Coordination bonds 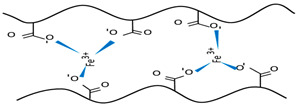	Adding divalent metal ions in some polymeric solutions causes coordination bonds between the biopolymer and metal ions, forming a hydrogel.	[92,93]
Colloidal assembly 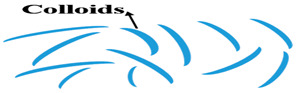	Specific polysaccharides, such as nanocellulose, have unusual self-assembling behavior. Nanocellulose particles exhibit fluid behavior in a diluted state, although they are gelled when the shear is removed.	[94]

**Table 5:** Preparation methods of chemical hydrogels.

	Methods	Explanation	Ref.
(1)	Polymerization in aqueous solution	It is a reaction between neutral and ionic monomers with a multifunctional cross-linking agent in a solvent, generally water or ethanol, a water–ethanol mixture, and benzyl alcohol. The product is washed with ethanol or distilled water to eliminate unreacted reagents and oligomers. The formed gel is dried, pulverized, and sieved to achieve a specific size.	[104,105]
	(1.a)-Radical polymerization	It is also called chain-growth polymerization or cationic or anionic polymerization. The process entails four steps: initiation, propagation, chain transfer, and termination. Water is most widely used as a solvent. This method includes graft polymerization.	
	(1.b)-Chemical reaction of functional groups	Cross-linking is performed by a reaction between functional groups (-COOH, -OH, -NH_2_) of hydrophilic polymers and polyfunctional cross-linking agents. As examples: (1.b.α), (1.b.β,) (1.b.γ), (1.b.δ), (1.b.ω), and (1.b.σ).	
	1.b.α-*Aldehydes:* Hydrophilic polymers with (–OH) form cross-links via aldehyde cross-linking agents, such as glutaraldehyde.		[106]
	(1.b). β-*Condensation reaction:* A reaction between -OH and COOH to form polyesters or between –NH_2_ and –COOH to form polyamides.		[107]
	(1.b).γ-*Addition reaction:* Where higher-functional cross-linkers react with functional groups of hydrophilic polymers (such as -OH, -NH_2_, and COOH).		[108]
	(1.b).δ-*Schiff-base reaction:* Occurs between aldehyde and amine groups. The gelation kinetics and the physical properties of SAP can be modified by changing the ratio of those groups.		[109,110,111,112]
	(1.b).ω-*Epoxide-based cross-linking:* Epoxide polymers and cross-linking agents (such as epichlorohydrin) are water-soluble compounds highly reactive to nucleophile groups of polysaccharides (-OH and -NH_2_).		[99,113]
	(1.b).σ-*Click chemistry:* Consists of three classical click reactions: Cu^2+^-catalyzed thiol-alkene addition, azide-alkyne (3 + 2) cycloaddition, and furan-maleimide (4 + 2) Diels–Alder cycloaddition.		[114]
	(1.c)-Enzyme-induced cross-linking	The SAP’s preparation is induced by enzymes (such as transglutaminase, tyrosinase, horseradish peroxidase, and lysyl oxidase) acting as a catalyst in cross-linking or forming covalent bonds with polysaccharide chains without interfering with other polymers’ functional groups.	[115]
(2)	Inverse-phase suspension polymerization	It involves two phases: - The organic phase consists of a non-polar solvent (such as toluene or n-hexane) and a stabilizer (to maintain the dispersion); - The aqueous phase consists of monomers, initiators, and cross-linker. The produced SAPs are obtained as powder or beads with desired sizes.	[116]
(3)	Irradiation polymerization	Irradiation is applied as an initiator to generate radicals’ formation on the polysaccharide chains (via homolytic splitting of the C-H bonds) for the cross-linkage action. It depends on various parameters, including radiation dose, the medium’s polymer concentration, and the presence of oxygen. The advantage of irradiation compared to the chemical initiation techniques is that the resulting hydrogel is relatively pure since no initiator is implicated. Commonly used methods are glow discharge [117], gamma-ray irradiation [118], electron beam irradiation [119], microwave irradiation, and ultrasonication [120].	[121,122,123]
(4)	Photo-polymerization	The cross-linking process uses a light corresponding to the absorption wavelength (180–220 nm) of the polysaccharide’s group and the cross-linking agent.	[124]

**Table 6:** Some recent polysaccharide-based composite hydrogels.

Materials	Synthesis	Results	Ref.
- Carboxymethyl cellulose (CMC). - Starch aldehydes (CS and PS, prepared by periodate oxidation (with NaIO_4_) of corn and potato starch). - Citric acid (CA).	Cross-linking reaction between CMC (1 g) and starch aldehyde (1 g) by CA (10% and 20% molar ratio).	- Application: water reservoir. - Porous structure with a large specific surface. - The highest equilibrium swelling ratios were 87.0 g/g and 80.6 g/g for CS20-CA0 and PS30-CA0, depending on the starch’s source and the cross-linking density.	[165]
- Sodium alginate (SA) (oxidized with NaIO_4_). - Chitosan oligosaccharide (COS). - Zinc oxide nanoparticles (ZnO NPs).	- SA + COS with different molar ratios (3:1, 2:1, 1:1, 1:2, and 1:3) to synthesize SA-COS hydrogels. - SA-COS-ZnO: mixing ZnO NPs (dispersed in 2 mg/mL of SA) with COS solution.	- Application: Wound healing. - 3D porous structure (80%). - Hydrogels provided a humid and antibacterial environment for wound healing, with good mechanical properties and swelling degree (maximum 150%). - 18% of Zn^2+^ was released in 24 h and 60% was released in 150 h. - Antibacterial activity followed the order SA-COS < SA-COS-ZnO, due to ZnO.	[166]
- Cellulose (pristine eucalyptus residues (PERs) or treated eucalyptus residues (TERs)). - Gelatin (G). - Glutaraldehyde (H) as a cross-linking agent.	- SAP GH: G cross-linked with H. - GH-PER, GH-TER (SAP composites) where TER and PER (1, 3, 5%) act as a filler (fibers).	- Application: Cr(VI) adsorption from contaminated water. - Fibers improved thermal stability, rigidity, and cross-linking density. - Maximum swelling capacity: 466.1%. - The swelling capacity followed the order: GH-PER_1_ (497.4%) > GH > GH-PER_3_ > GH-PER_5_ and GH > GH-TER_1_ (413.9%) > GH-TER_3_ > GH-TER_5_. - The adsorption capacity followed the order: GH-TER_5_ (13.3) > GH-PER_3_, GH-PER_5_, GH-TER_3_ (12.4) > GH (12.3) > GH-TER_1_ (12.2) > GH-PER_1_ (12).	[167]
- Cellulose. - Chitosan. - LiBr (solvent).	Via a codissolution and regeneration procedure in LiBr, with different ratios of cellulose and chitosan	- Application: removal of heavy metals (Cu^2+^, Zn^2+^, and Co^2+^) from water. - Chitosan introduced functionality for metal adsorption, increased the specific surface area, and enhanced the mechanical strength (due to H-bonds) of the composite SAP. - Mesoporous structure (27–300 Å). - Metal adsorption followed the order: Cu^2+^ > Zn^2+^ > Co^2+^.	[168]
- N-carboxymethyl chitosan (CMC). - Alginate (Alg). - Calcium chloride (CaCl_2_) as a cross-linking agent.	Dual-physical cross-linking via both electrostatic interaction and divalent chelation by Ca^2+^ cations with various compositions.	- Application: Cell proliferation and wound healing. - Enhanced mechanical properties. - 3D network structure with irregular pores (dimeter = 50–100 µm). - CMC-Alg-4, prepared with 1 g of CMC, 40 mg of Alg, and 32 mg of CaCl_2_, exhibited better results in terms of water retention ability, rheology, the release rate of EGF, cell proliferation efficiencies, and wound healing.	[169]
- Chitosan (CS). - Carboxymethyl cellulose (CMC). - Graphene oxide (GO) as a cross-linking agent. - Potassium persulfate (KPS) as initiator.	CS (0.5 g) and CMC (0.5 g) are chemically cross-linked by GO, which was previously functionalized with vinyl groups via grafting with VTES.	- Application: Adsorption of dyes *(cationic MB and anionic MO)* from contaminated water. - Adsorption of 82% dye (from 50 mg/L of MO solution) with 0.4 g/L of the hydrogel at pH 3 and 99% dye (from 50 mg/L of MB solution) with 0.4 g/L of the hydrogel at pH 7. - Maximum adsorption capacities: 404.52 mg/g for MO and 655.98 mg/g for MB.	[170]
- Cellulose nanofibers (CNFs). - Starch (ST). - Poly (acrylic acid) (PAA). - Potassium persulfate (KPS) as initiator. - MBA as a cross-linking agent.	CNFs incorporated in different compositions in ST-g-PAA, previously prepared by graft polymerization in the presence of KPS and MBA.	- Application: Removal of Cu^2+^ ions from water. - Cu^2+^ adsorption capacity of SAPs was improved after the incorporation of NFCs. - Maximum Cu^2+^ uptake was 0.957 g/g in 0.6 g/L Cu^2+^ solution at pH (5).	[171]
- Magnetic nanocellulose (m-CNCs) (coprecipitated from cellulose nanocrystals. - Alginate (Alg). - CaCl_2_ for physical cross-linking.	m-CNCs were incorporated into alginate-based hydrogel beads, physically cross-linked with CaCl_2_.	- Application: Drug delivery (ibuprofen). - The highest swelling degrees were 2477% in PBS medium, 515% in SGF, and 665% in water. - m-CNCs improved the mechanical toughness, increased the swelling rates, and decreased the rate of drug release of the SAPs.	[172]
- 2,3-dialdehyde cellulose (DAC) (by periodate oxidation of nanocellulose). - Chitosan (CS).	Cross-linking between DAC and CS *(dissolved in HCl)* with different compositions at three different reaction temperatures (22.5, 40, and 80 °C).	- Application: Adsorption of Congo red dye. - The SAPs had a porous structure and showed good thermal and morphological stability, with a fast and high adsorption rate at pH 2 (a maximum of 100%) and excellent desorption properties at pH 12.	[173]
- Cellulose. - Chitosan. - Dialdehyde cellulose (DAC) as a cross-linking agent. - LiOH and urea as solvents.	Via dissolution–regeneration by LiOH/urea aqueous solution, before cross-linking reaction with DAC *(Schiff base reaction with chitosan)*, with various compositions.	- Application: Adsorption of bovine serum albumin (BSA). - Good thermal stability, with higher stability in pH 2–9 over 21 days. - The higher BSA adsorption was about 470 mg/g at pH 5.5, due to the significant electrostatic interactions between protonated amino groups of chitosan and the dissociated carboxyl groups of BSA.	[174]

**Table 7:** Gel content variation of some bio-based hydrogels.

SAP Based on	Hydrogels	Gel Content Variation	Ref.
- Chitosan (CS) - Na-alginate (Alg) - Polyacrylamide (PAAm)	Via γ-rays: - PAAm-Alg - PAAm-Alg-CS - PAAm-CS; with several copolymer compositions.	- For PAAm-Alg: Any increase in Alg content or decrease in irradiation dose causes a reduction of the gel content. - For PAAm-CS: The gel content decreases with an increase in irradiation dose or chitosan content. - For PAAm-Alg-CS: At lower irradiation doses, similar behavior of PAAm-Alg was obtained. The gel content decreases in this order: PAAm-Alg > PAAm-Alg-CS > PAAm-CS.	[191]
- Sodium carboxymethyl cellulose (NaCMC) - FeCl_3_	Using different percentages of the reagents.	When the concentration of NaCMC increases, the cross-linking density increases, so the gel content increases. *NaCMC-12*, prepared by NaCMC (7%) and FeCl3 (10%), presents the full gel content.	[192]

**Table 8:** Factors of saline solutions affecting swelling capacity.

Effect of:	Swelling Behavior	Ref.
Salt concentration	Increasing the ionic concentration reduces the mobile ion concentration between the hydrogel network and the external medium (i.e., osmotic swelling pressure), reducing the hydrogel volume and the gel shrinks. As a result, the swelling rate decreases.	[182,191,222,230,231]
Charge	Hydrogels with carboxylic moieties have varying swelling capacities in mono-, di-, and trivalent cation solutions. The hydrogel swelling is compared in monovalent (NaCl), divalent (CaCl_2_), and trivalent (AlCl_3_) ions at 0.5 M in solution at 25 °C. Multivalent cations (Ca^2+^ and Al^3+^) create coordination complexes with -COO^−^ groups of SAP. These interactions serve as further cross-linkages in the gel network, significantly reducing the water absorption rate. In fact, trivalent cations reduce the absorption capacity more than bivalent cations, which are more effective than monovalent cations. So, when the cation charge increases (Na^+^ < Ca^2+^ < Al^3+^), the absorption capacity decreases following the order Al^3+^ < Ca^2+^ < Na^+^.	
Ion size	The SAP’s capacity to absorb water increases with decreasing radius of the cation of the same valence. The results of this factor are useful because as the size of the ions in the swelling media increases (e.g., Na^+^ < K^+^ and Mg^2+^ < Ca^2+^), the swelling capacity of the hydrogel decreases (following the order Na^+^ > K^+^ and Mg^2+^ > Ca^2+^), due to the difficult penetration of the ions into the SAP.	

**Table 9:** Adsorption of heavy metals, dyes, and other pollutants using polysaccharide-based adsorbents.

	Polysaccharides Used in Preparing Adsorbent Hydrogels	Heavy Metals or Dyes	Maximum Adsorption Capacity in mg/g	Ref.
Heavy metals	Chitosan	Cu(II), Cr(VI)	116.6 and 107.5, respectively	[250]
	Chitosan	Cu(II)	185.5	[251]
	Chitosan/Alginate	Pb(II), Cd(II), and Cu(II)	176.5, 81.25, and 70.83, respectively	[252]
	Chitosan	Cr(VI)	102.56	[253]
	Cellulose	Pb^2+^	558.7	[254]
	Cellulose	Cu(II), Ni(II), Zn(II), Pb(II), and Cr(III)	253.8, 112.2, 148.4, 248.2, and 30.4, respectively	[255]
	Cellulose	Ni(II) and Cu(II)	112.74 and 109.77, respectively	[256]
	Chitosan/Starch	Cu^2+^, Ni^2+^, Co^2+^	100.6, 83.25, and 74.01, respectively	[49]
	Chitosan/Glucan	Pb(II), Cu(II), Cd(II), Co(II), and Ni(II)	395, 342, 269, 232, and 184, respectively	[139]
	Cellulose	Cr(VI)	13.3	[167]
	Cellulose/Chitosan	Cu^2+^, Zn^2+^, Co^2+^	Cu^2+^ > Zn^2+^ > Co^2+^, where Cu^2+^ (94)	[168]
	Cellulose nanofibers/Starch	Cu^2+^	957	[171]
	Alginate	Cu^2+^, Cd^2+^	13.38 and 9.54, respectively	[257]
	Alginate	Pb^2+^	234.8	[258]
	Guar gum	Cr^6+^	101	[259]
	Pectin	Pb^2+^	390.9	[260]
	Salecan	Cd^2+^	421.5	[261]
	κ-Carrageenan	Hg^2+^	229.9	[262]
Dyes	Chitosan	Methyl orange	1060	[263]
	Chitosan	Methylene blue	20.408	[264]
	Chitosan/β-Cyclodextrin	Reactive blue 49	498	[265]
	Starch	Methylene blue	2276	[266]
	Starch	Methylene blue	2225	[267]
	Alginate/Chitosan	Methylene blue	137.2	[268]
	Xanthan gum	Crystal violet	1567	[269]
	Agarose/κ-Carrageenan	Methylene blue	242.3	[270]
Heavy metals and dyes	Cellulose	Cu(II), methylene blue	85 and 138, respectively	[128]
	Chitosan	Cd(II), methylene blue	90.038 and 23.478, respectively	[141]
	Starch	Cr(VI), naproxen drug	420.13 and 309.82, respectively	[157]
	Starch	Co^2+^, basic violet	350 and 600, respectively	[271]
	Pectin	Methyl violet, methylene blue, Pb(II), Cu(II), Co(II), and Zn(II)	265.49, 137.43, 54.86, 53.86, 51.72, and 50.01, respectively	[187]
Other pollutants	Alginate	Phenol	994	[272]
	Cellulose	Tetracycline	44.9	[273]
	Alginate	Phosphate	16.4	[274]
	Konjac glucomannan	Phosphate	16.1	[275]
	Chitosan	Ciprofloxacin	82	[276]
	Agarose	Ofloxacin	581.4	[277]
	Xanthan gum	Bisphenol A	458	[278]

**Table 10:** Examples of polysaccharide-based SAPs for biomedical and pharmaceutical applications.

	Polysaccharides Used in Hydrogel Preparation	Applications	Ref.
Drug delivery	Cellulose	Drug delivery	[282]
	Carboxymethyl cellulose	Drug release in cancer therapy	[125]
	Carboxymethyl cellulose	Drug delivery	[130]
	Chitosan/Dialdehyde starch	Betamethasone ocular delivery	[283]
	Carboxymethyl chitosan/Alginate	Lidocaine delivery	[284]
	Chitosan/Pullulan	Ibuprofen, bacitracin, and neomycin delivery	[285]
	Nanocellulose/Alginate	Ibuprofen delivery	[172]
Wound dressing	Carboxymethyl cellulose	Dressing and skin replacement	[204]
	Sodium alginate/Chitosan	Wound healing	[166]
	Carboxymethyl cellulose/Alginate	Cell proliferation and wound healing	[169]
	Carboxymethyl chitosan/Methacrylate sodium alginate	Skin wound healing	[286]
Tissue engineering	Alginate	Meniscal repair	[181]
	Alginate	Cartilage tissue engineering	[287]
	Cellulose nanofibers/Chitosan	Intervertebral disc annulus fibrosus tissue repair	[288]
	Chitosan	Cartilage tissue engineering	[289]

## Data Availability

The datasets used and/or analyzed during the current study are available from the corresponding author upon reasonable request.
